# Ldha Regulates Osteosarcoma Lung Metastasis through Hedgehog Signaling

**DOI:** 10.1158/2767-9764.CRC-25-0163

**Published:** 2026-06-25

**Authors:** Rie Maki, Shingo Komura, Akihito Nagano, Ayumi Niwa, Hiroyuki Tomita, Yuuki Imai, Haruhiko Akiyama

**Affiliations:** 1 Department of Orthopaedic Surgery, Gifu University Graduate School of Medicine, Gifu, Japan.; 2 Department of Tumor Pathology, Gifu University Graduate School of Medicine, Gifu, Japan.; 3 Center for One Medicine Innovative Translational Research (COMIT), Gifu University, Gifu, Japan.; 4 Division of Integrative Pathophysiology, Proteo-Science Center, Ehime University, Toon, Japan.

## Abstract

Osteosarcoma is the most common malignant bone tumor in children and adolescents, with a high propensity for lung metastasis, leading to poor prognosis. Lactate dehydrogenase A (LDHA), a key glycolytic enzyme, has been implicated in the progression and metastasis of various cancers. In this study, we demonstrated that *Ldha* knockdown reduced cell migration and invasion in murine osteosarcoma cells and decreased lung metastasis. RNA sequencing analysis revealed that *Ldha* knockdown was associated with the downregulation of Hedgehog signaling. Consistently, pharmacologic inhibition of Hedgehog signaling using vismodegib suppressed cell migration, invasion, and lung metastases in murine osteosarcoma cells. In human osteosarcoma cells, *LDHA* silencing downregulated Hedgehog signaling, and inhibition of Hedgehog signaling suppressed cell migration and invasion. These findings highlight the involvement of the Ldha–Hedgehog signaling axis in osteosarcoma lung metastases, providing insights into its molecular mechanisms and potential therapeutic targets.

**Significance:**

*Ldha* knockdown downregulates Hedgehog signaling and inhibits osteosarcoma lung metastasis, presenting a potential therapeutic target for osteosarcoma.

## Introduction

Osteosarcoma is a primary malignant bone tumor that primarily affects children and adolescents. Advances in standard treatment, including neoadjuvant and adjuvant chemotherapy along with surgery, have improved the 5-year survival rate to ∼70% (1). However, 15% to 20% of the patients present with clinically detectable metastases at diagnosis, and those with metastatic or recurrent disease have a poor prognosis, with a 5-year survival rate of ∼20%. The lungs are the most common site of metastasis (2, 3). Therefore, preventing lung metastasis is a critical aspect of osteosarcoma treatment, and there is an urgent need to (i) identify novel therapeutic strategies to inhibit metastasis and (ii) achieve local tumor control.

Glycolysis is upregulated in various human cancers, even in the presence of sufficient oxygen, and increased energy metabolism through glycolysis is a hallmark of cancer (4, 5). In addition, upregulated glycolysis provides intermediates, such as amino acids, nucleotides, and lipids, that feed into biosynthetic pathways. In cancer cells, glycolysis plays a crucial role in maintaining redox homeostasis to overcome oxidative stress (6–10). Lactate dehydrogenase A (LDHA), which catalyzes the conversion of pyruvate and NADH to lactate and NAD^+^, is a key component of glycolysis. High LDHA expression has been reported in many cancers and is associated with poor prognosis (11–17). Enhanced glycolysis supports the high energy demands of rapidly proliferating cancer cells while supplying essential metabolites for cancer cell survival, proliferation, migration, and invasion. Furthermore, lactate produced by LDHA contributes to tumor progression by modifying the tumor microenvironment to promote cancer cell survival and evade immune surveillance (18, 19). *LDHA* knockdown reduces tumor cell proliferation *in vitro* in cancers such as cervical cancer (13), pancreatic cancer (14), and gastric cancer (17). LDHA inhibition suppresses tumor growth (15, 16) and enhances sensitivity to radiotherapy or chemotherapy both *in vitro* and *in vivo* (20–22). Targeting LDHA reduces metastasis in pancreatic adenocarcinoma (12) and papillary thyroid carcinoma (23). These findings indicate that *LDHA* plays a crucial role in tumor growth and metastasis in various human cancers and is a potential therapeutic target.

In recent years, the role of LDHA in osteosarcoma has been investigated. Inhibition of LDHA decreased cell proliferation and invasion in human osteosarcoma cells, and silencing of *LDHA* impaired tumorigenesis *in vivo* (24). Chen and colleagues (25) identified LDHA as a downstream target of miR-323a-3p, demonstrating that overexpression of miR-323a-3p reduces LDHA expression and inhibits osteosarcoma cell proliferation. miR-329-3p targets *LDHA* and is involved in cisplatin resistance (26). Cui and colleagues (27) reported that 2-((3-cyanopyridin-2-yl)thio)acetamides, identified as LDHA inhibitors, suppress MG-63 proliferation and induce apoptosis. Furthermore, oncogenic circRNA, Circ_0000376, regulates the miR-577–HK2–LDHA signaling pathway to promote the growth and invasion of and glycolysis in osteosarcoma (28). According to Mei and colleagues (29), in osteosarcoma cells, LDHA expression is regulated by epigenetic modifications mediated by NAT10, ac4C-YTHDC1, and m6A. These studies suggest that LDHA regulates osteosarcoma proliferation and invasion. However, few studies have investigated the role of LDHA in osteosarcoma metastasis. Shen and colleagues reported that upregulated KDM6B promotes metastasis in osteosarcoma by modifying LDHA expression (30). Therefore, the molecular mechanisms by which LDHA affects osteosarcoma progression, particularly its impact on metastasis, are underexplored.

In this study, we examined the effects of LDHA inhibition on osteosarcoma cells both *in vitro* and *in vivo* and found that although *LDHA* knockdown suppressed lung metastasis, it did not affect local tumor growth. Further analysis revealed that LDHA regulates Hedgehog signaling, which plays a role in osteosarcoma lung metastasis.

## Materials and Methods

### Cell culture

Murine osteosarcoma LM8 cells and human osteosarcoma SaOS-2 and NOS1 cells were obtained from the RIKEN BRC Cell Bank (cat. #RCB1450, RRID: CVCL_6669, cat. #RCB0428, RRID: CVCL_0548, cat. #RCB1032, RRID: CVCL_1610). Human osteosarcoma HOS, MG63, and U2-OS cells were purchased from the American Type Culture Collection (ATCC; cat. #CRL-1543, RRID: CVCL_0312, cat. #CRL-1427, RRID: CVCL_0426, cat. #HTB-96, RRID: CVCL_0042). LM8 cells were cultured in Minimum Essential Medium-α (MEM-α, Thermo Fisher Scientific, cat. #12571063) supplemented with 10% fetal bovine serum (FBS, BioWest, cat. #S1810) and 100 U/mL penicillin–streptomycin (Wako, cat. #16823191). NOS1 cells were maintained in RPMI 1640 (Gibco, cat. #11875093) containing 10% FBS and 100 U/mL penicillin–streptomycin. Human osteosarcoma cell lines (SaOS-2, HOS, MG63, and U2-OS) were maintained in Dulbecco’s modified Eagle medium (Wako, cat. #04429765) containing 10% FBS and 100 U/mL penicillin–streptomycin. All cell lines were authenticated by the RIKEN BRC or ATCC using short tandem repeat profiling and were tested for *Mycoplasma* contamination using VenorGeM OneStep (Minerva Biolabs, cat. #11-8025), and cells were not maintained beyond 10 passages following thawing from master stocks. All cell lines were cultured at 37°C in a humidified atmosphere with 5% CO_2_. Vismodegib (Chemscene, cat. #CS-0255) and purmorphamine (Selleckchem, cat. #S3042) were used to inhibit and activate Hedgehog signaling pathway, respectively.

### Cell proliferation assay

Cell proliferation was assessed using Cell Counting Kit-8 (CCK-8, Dojindo, cat. #34707621) following the manufacturer’s instructions. Briefly, 1 × 10^4^ cells were seeded in a 96-well plate and incubated with inhibitors for 48 hours. Then the cells were incubated with CCK-8 reagent for 2 hours at 37°C. Absorbance was measured at 450 nm using Model 680 Microplate Reader (Bio-Rad).

### Establishment of *Ldha* stable knockdown murine osteosarcoma cell line

For short hairpin (sh) RNA transfection, shRNA plasmids (LDH-A shRNA plasmid, sc-45898-SH; control shRNA plasmid-A, sc-108060) were obtained from Santa Cruz Biotechnology and transfected according to the manufacturer’s instructions. Briefly, LM8 cells were seeded into a six-well plate and incubated until they reached ∼50% confluency. The next day, 1 mL of shRNA transfection medium containing 1 μg of shRNA plasmid was added to the cells. The plasmid was gently mixed and incubated with the cells for 5 hours. Then the cells were mixed and incubated with 1 mL of MEM-α supplemented with 20% FBS and 200 U/mL penicillin–streptomycin for 48 hours.

To select stable transformants, the cells were mixed with fresh MEM-α containing puromycin (5 μg/mL). The medium was replaced every 3 days with fresh antibiotics until resistant colonies were established. The selected colonies were expanded and validated by quantitative real-time PCR (qPCR) and Western blotting to confirm *Ldha* knockdown.

### siRNA transfection

*LDHA*-specific siRNA (siLDHA) and negative control siRNA were purchased from Dharmacon (ON-TARGET plus Human LDHA, cat. #L-008201-00-0005, ON-TARGETplus Nontargeting Control Pool, cat. #D-001810-10-05). Targeting sequences for *LDHA* were a pool of the following four target sequences: sequence 1, GGA​GAA​AGC​CGU​CUU​AAU​U; sequence 2, GGC​AAA​GAC​UAU​AAU​GUA​A; sequence 3, UAA​GGG​UCU​UUA​CGG​AAU​A; and sequence 4, AAA​GUC​UUC​UGA​UGU​CAU​A. Target sequences for the nontargeting pool were as follows: sequence 1, UGG​UUU​ACA​UGU​CGA​CUA​A; sequence 2, UGG​UUU​ACA​UGU​UGU​GUG​A; sequence 3, UGG​UUU​ACA​UGU​UUU​CUG​A; and sequence 4, UGG​UUU​ACA​UGU​UUU​CCU​A. Cells were transfected with siRNA oligonucleotides using Lipofectamine RNAiMAX Reagent (Invitrogen, cat. #13778150) following the manufacturer’s instructions. After 48 hours of incubation, knockdown efficiency was assessed by qPCR.

### RT-PCR and qPCR

Total RNA was extracted from cultured cells using RNeasy Plus Mini Kit (Qiagen, cat. #74136). Complementary DNA was synthesized using PrimeScript RT Master Mix (Takara, cat. #RR036B). RT-PCR was performed with AmpliTaq Gold 360 Master Mix (Thermo Fisher Scientific, cat. #4398881). The PCR products were separated on 2% agarose gels, stained with ethidium bromide, and visualized under ultraviolet light. qPCR was conducted using TB Green Premix Ex Taq II (Takara, cat. #RR820W). Relative mRNA expression levels were determined using the ΔΔCt method, with β-actin and β2-microglobulin as normalization controls. Primer sequences are listed in Supplementary Table S1.

### Wound healing assay

LM8 cells were seeded in 12-well plates at a density of 1 × 10^5^ cells per well and cultured until confluence. Before creating the scratch, the cells were treated with 5 μg/mL mitomycin C (KYOWA KIRIN) for 2 hours to inhibit proliferation. A 1,000-μL pipette tip was used to create a scratch. The wells were gently washed with PBS two times and treated with the specified reagent concentrations. Cell migration was monitored at 0, 24, 48, and 72 hours, and images were captured at each time point. The percentage of wound healing was calculated using the following formula: wound area at 0 hour − wound area at 24, 48, or 72 hour/wound area at 0 hour × 100. All experiments were performed in triplicate.

### Cell migration and invasion assays

Cell migration and invasion were assessed using a Transwell chamber assay (8-μm pore size, 24-well plate) with or without Matrigel coating (Corning Incorporated, cat. #354480, #3422). For the migration assay, 5 × 10^4^ cells suspended in serum-free MEM-α were seeded into the upper chamber, and 500 μL of MEM-α containing 10% FBS were added to the lower chamber.

For the invasion assay, 5 × 10^4^ cells in serum-free MEM-α were seeded into the upper chamber precoated with Matrigel, and 500 μL of MEM-α containing 10% FBS were added to the lower chamber.

After 24 hours (for the migration assay) and 48 hours (for the invasion assay), the medium was removed, and the cells were fixed with 4% paraformaldehyde for 15 minutes and stained with 0.1% crystal violet for 10 minutes. The number of migrated or invaded cells was counted in five randomly selected fields at 200× magnification using a BX51 microscope (Olympus).

### Measurement of lactate production

Lactate levels in the culture medium were measured using Glycolysis Cell-Based Assay Kit (Cayman Chemical, cat. #600450) following the manufacturer’s instructions.

Briefly, 10 μL of the supernatant from each cell culture well was transferred to a 96-well plate containing 90 μL of assay buffer. Next, 100 μL of reaction solution was added to each well, and the plate was incubated on an orbital shaker at room temperature for 30 minutes. Absorbance (OD value) was measured at 490 nm using Model 680 Microplate Reader (Bio-Rad). Normalization was performed by cell number.

### RNA sequencing analysis

Total RNA was extracted using ISOGEN Reagent (Nippon Gene, cat. #31102501) and RNeasy Plus Mini Kit (Qiagen). The integrity of the isolated RNA was assessed using 2100 Bioanalyzer Instrument (Agilent Technologies). RNA sequencing (RNA-seq) libraries were prepared using NEBNext Ultra II Directional RNA Library Prep Kit for Illumina (New England Biolabs). Sequencing was performed using NextSeq 500 System (Illumina, RRID: SCR_014983) and NextSeq 500/550 High Output Reagent Kit v2.5 (75 Cycles), generating 75-base pair paired-end reads. Differential gene expression analysis was conducted using an exact test following normalization. Pathway analyses were performed using iDEP.951 as described (31).

### Western blotting assay

Cells were collected and lysed using RIPA buffer supplemented with Halt Protease and Phosphatase Inhibitor Cocktail (Thermo Fisher Scientific, cat. #78440). Protein concentrations were determined using Protein Assay BCA Kit (Wako, cat. #29773101). Proteins were separated by SDS-PAGE using NuPAGE 10% Bis-Tris, 1 mm Mini Protein Gels (Thermo Fisher Scientific, cat. #NP0303BOX) and subsequently transferred to membranes. The membranes were blocked with a reagent containing 2% skim milk (Wako, cat. #19810605) for 1 hour at room temperature, incubated with primary antibodies overnight at 4°C, and incubated with secondary horseradish peroxidase (HRP)-linked antibodies for 1 hour at room temperature. Blots were visualized using ImageQuant LAS4000 mini (Cytiva) and quantified with ImageJ software. Band intensities were normalized to β-actin and plotted on a graph. The primary antibodies used included anti-LDHA (Cell Signaling Technology, #2012, RRID: AB_2137173, 1:1,000), anti-Gli1 (Cell Signaling Technology, #3538, RRID: AB_1903989, 1:1,000, Santa Cruz, #sc-515781, 1:500), anti-Patched/PTCH1 (Santa Cruz, #sc-518102, 1:100, abcam, ab53715, RRID: AB_882208, 1:1,000), anti-smoothened (Smo; abcam, ab236465, RRID: AB_2935839, 1:1,000), and anti–β-actin (Cell Signaling Technology, #4970, RRID: AB_2223172, 1:3,000).

### Animal experiments

All animal experiments were conducted with approval from the Gifu University Animal Experiment Committee (2021-279, 2022-120) and adhered to the *Animal Research: Reporting in Vivo Experiments* guidelines.

For the tumorigenesis and spontaneous metastasis assays, LM8-shCTL, -shLdha1, or -shLdha2 cell suspensions (1 × 10^6^ cells/100 μL PBS) were mixed with 100 μL Matrigel (Corning, cat. #356234) and subcutaneously injected into the right flank of six-week-old female C3H mice (The Jackson Laboratory, RRID: MGI:2161025) under isoflurane anesthesia. To assess the antimetastatic effect of vismodegib, LM8 osteosarcoma cells were subcutaneously implanted into six-week-old female C3H mice. The mice were randomly assigned to either the treatment group or control group (*n* = 10 per group). The treatment group received 50 mg/kg vismodegib orally; the control group was given DMSO (Wako, cat. #04307216) for 6 days a week, starting the day after tumor cell inoculation and continuing until the study endpoint. In both the LDHA inhibition and vismodegib administration experiments, body weight and tumor volume were measured two times weekly. Tumor volumes were calculated using the following formula: tumor volume = (width^2^ × length)/2. After 28 days of monitoring, tumors and lungs were harvested. The lungs were fixed with Bouin’s solution (Sigma Aldrich, cat. #HT10132) to quantify visible metastatic tumor nodules. Bouin-fixed, paraffin-embedded lung tissue sections (4-μm-thick) were prepared, with four slides collected per lung and stained using hematoxylin and eosin. The stained slides were scanned, and metastatic tumor nodules and areas were quantified. First, the area of the entire lung in the section was measured, followed by the areas of the metastatic regions. The percentage of metastatic areas to total lung area was determined to evaluate metastasis. All measurements were done by using ImageJ (NIH software, RRID: SCR_003070). To account for potential variability associated with oral administration of vismodegib, the highest and lowest values in each group were excluded as potential outliers, resulting in eight samples per group for analysis.

### Clinical samples and immunohistochemistry

The use of human osteosarcoma clinical samples was approved by the Ethics Committee of Gifu University (Approval No. 2023-275), and all ethical guidelines for research involving human participants were followed. Osteosarcoma tissue samples were obtained from 19 patients admitted to Gifu University Hospital between 2006 and 2021. The specimens were fixed in 4% paraformaldehyde and embedded in paraffin for immunohistochemical (IHC) analysis. Sections were cut into 4-μm-thick slices and deparaffinized using xylene and ethanol. Antigen retrieval was performed by incubating the sections in sodium citrate buffer (pH 6.0) at 95°C for 10 minutes. Endogenous peroxidase activity was blocked with 3% H_2_O_2_ for 10 minutes at room temperature, followed by two 5-minute washes with PBS. The tissue sections were incubated overnight with primary antibodies, including anti-Gli1 (Cell Signaling Technology, #3538, RRID: AB_1903989, 1:300) and control IgG (Cell Signaling Technology, #3900, RRID: AB_1550038, 1:300). The tissue sections were washed two times with PBS and treated with an HRP-labeled anti-rabbit secondary antibody. The reaction products were visualized using 3,3′-diaminobenzidine tetrahydrochloride (Dako, cat. #K3467) and counterstained with hematoxylin. Histologic scoring was performed independently by two pathologists in a blinded manner.

### Lactate treatment

Sodium L-lactate (Sigma Aldrich, cat. #71718-10G) or L-(+)-lactic acid (Sigma Aldrich, cat. #L1750-10G) was added the day after cells were seeded and used for further experiments.

### Measurement of reactive oxygen species

Reactive oxygen species (ROS) levels were evaluated using Highly Sensitive DCHF-DA ROS Assay Kit (Dojindo, #340-09811) according to the manufacturer’s instructions. Briefly, cells were cultured in chamber slides at density of 5 × 10^4^ cells/chamber overnight. Then, cells were incubated with Highly Sensitive DCFH-DA Dye working solution at 37°C, 5% CO_2_ for 30 minutes. Cells were visualized through microscopy (BX51, Olympus). For quantification, 1 × 10^4^ cells/well were seeded in a flat 96-well black clear-bottom plate with lid (Costar, #3603), and fluorescence was measured using EnVision (Perkin Elmer, #2104-0020) with excitation and emission wavelengths of 485 and 520 nm, respectively.

### Statistical analysis

Statistical analysis was conducted using a two-tailed Student *t* test, one-way ANOVA, or Mann–Whitney *U* test. Data were plotted using GraphPad PRISM software version 10.4.1 (GraphPad, Inc. RRID: SCR_002798) and presented as the mean ± SEM. Statistical significance was defined as *P* < 0.05.

## Results

### Genetic silencing of *Ldha* inhibits proliferation, migration, and invasion of osteosarcoma cells *in vitro*

**Figure 1.**
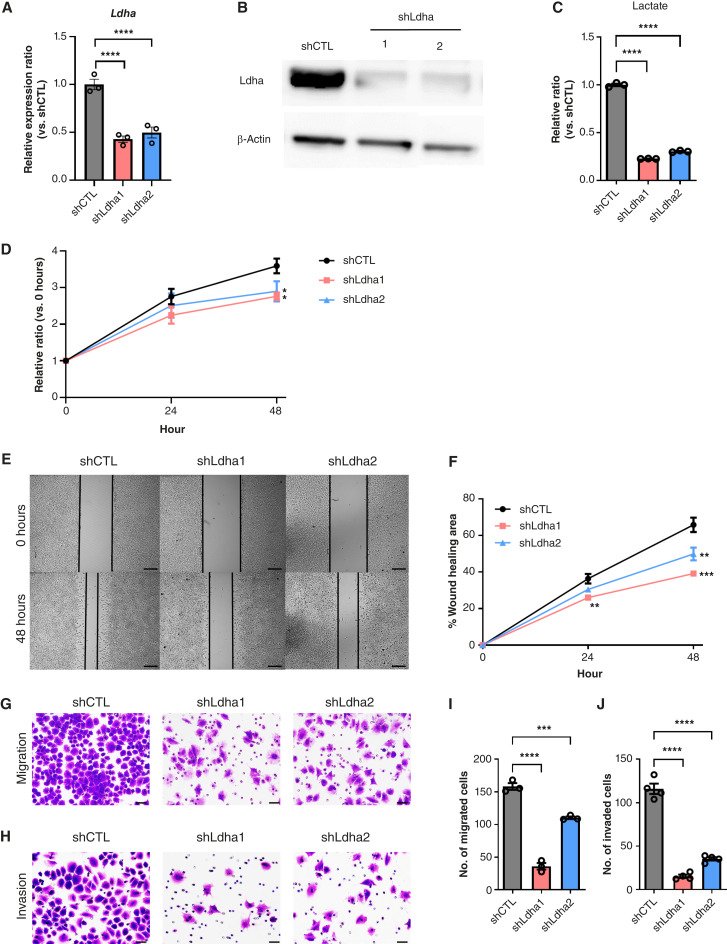
*LDHA* knockdown suppresses the proliferation, migration, and invasion of murine LM8 osteosarcoma cells. **A,** Expression of *Ldha* mRNA in LM8 cells following transfection with *Ldha* or control (CTL) shRNA (*n* = 4; independent experiments). The expression level of shCTL was set to 1. **B,** Protein expression of LDHA in LM8 cells after transfection with *Ldha* or CTL shRNA. **C,** Lactate production in LM8-shCTL, shLdha1, and shLdha2 cells (*n* = 4; independent experiments). The expression level of shCTL was set to 1. **D,** Cell proliferation assay of LM8-shCTL, shLdha1, and shLdha2 cells was assessed using the CCK-8 assay (*n* = 8; independent experiments). Absorbance at 0 hour was set to 1. **E,** Wound healing assay to evaluate the migration capacity of LM8-shCTL, shLdha1, and shLdha2 cells. Scale bars, 200 μm. **F,** Quantification of wound healing rate in (**E**; *n* = 4; independent experiments). **G,** Transwell migration assay (without Matrigel) to assess migration capacity in LM8-shCTL, shLdha1, and shLdha2 cells. Scale bars, 50 μm. **H,** Transwell invasion assay (with Matrigel) to evaluate invasion capacity in LM8-shCTL, shLdha1, and shLdha2 cells. Scale bars, 50 μm. **I,** Quantification of migration ability from (**G**), showing the number of migrated cells after 24 hours (*n* = 3; independent experiments). **J,** Quantification of invasion ability from **H**, showing the number of invaded cells after 48 hours (*n* = 4; independent experiments). Data are presented as the mean ± SEM. *P* values were calculated using one-way ANOVA test (**A**, **C**, **D**, **F**, **I**, and **J**). *, *P* < 0.05; **, *P* < 0.01; ***, *P* < 0.001; and ****, *P* < 0.0001 vs. control.

To investigate the role of Ldha in osteosarcoma, we established osteosarcoma cell lines with stable *Ldha* knockdown using shRNA in the highly metastatic murine osteosarcoma cell line LM8 (32). We generated two LM8 cell lines with stable Ldha suppression—shLdha1 and shLdha2. RT-qPCR and Western blot analysis confirmed a significant reduction in Ldha expression in shLdha1 and shLdha2 cells compared with shCTL cells (Fig. 1A and B). shLdha1 and shLdha2 cells exhibited decreased lactate production, indicating reduced Ldha activity in the knockdown cells (Fig. 1C). We assessed whether *Ldha* knockdown affected osteosarcoma cell proliferation, migration, and invasion. The CCK-8 assay revealed a significant reduction in the proliferation of shLdha cells compared with shCTL cells (Fig. 1D). In the wound healing assay, *Ldha* knockdown markedly impaired the migration ability of LM8 osteosarcoma cells (Fig. 1E and F). Transwell migration and invasion assays demonstrated that both migratory and invasion capabilities were diminished in shLdha1 and shLdha2 cells (Fig. 1G–J). These findings suggest that LDHA plays a critical role in the proliferation, migration, and invasion of LM8 osteosarcoma cells.

### Genetic silencing of *Ldha* suppresses lung metastasis in osteosarcoma cells *in vivo*

**Figure 2.**
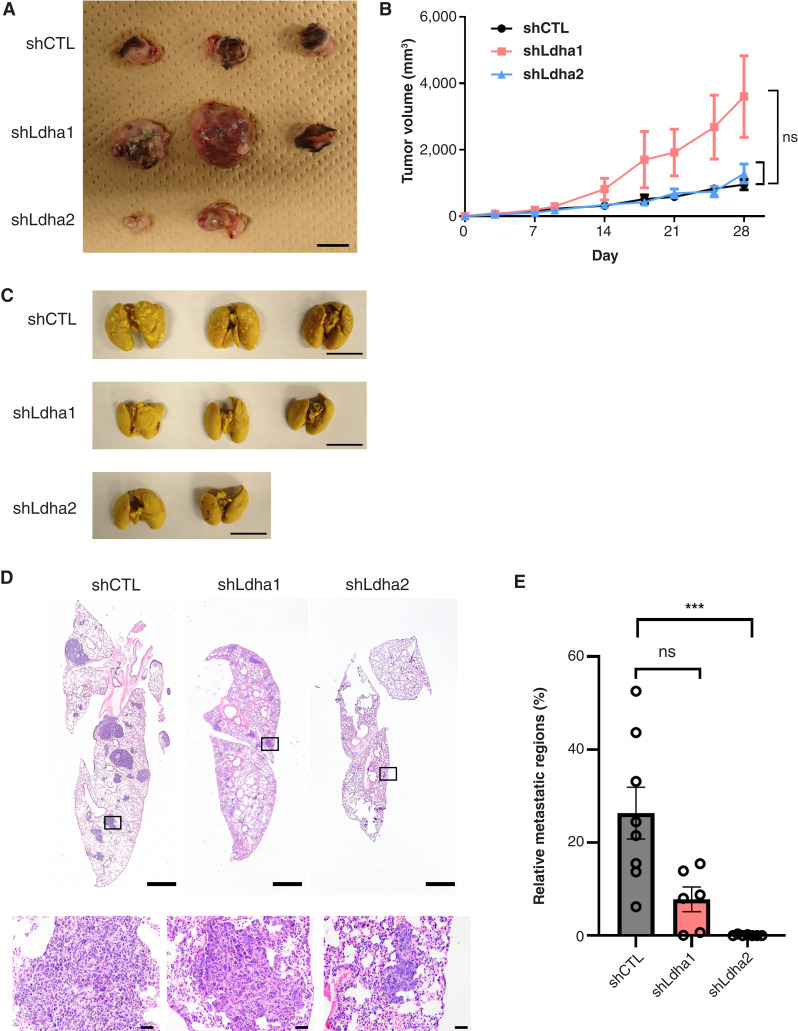
*LDHA* knockdown inhibits lung metastasis of murine osteosarcoma *in vivo*. **A,** Representative tumor images from mice subcutaneously implanted with LM8-shCTL, shLdha1, and shLdha2 cells. Scale bars, 10 mm. **B,** Tumor growth curves based on volume measurements at the indicated time points (*n* = 6–8 biological replicates per group). **C,** Bouin’s solution–fixed lung tissues from mice bearing LM8-shCTL, shLdha1, and shLdha2 cell–derived tumors. Scale bars, 10 mm. **D,** Hematoxylin and eosin–stained lung sections from the shCTL, shLdha1, and shLdha2 groups. The top row at low magnification (scale bars, 1,000 μm) and the bottom row at high magnification (scale bars, 50 μm). **E,** Quantification of the metastatic area relative to total lung area in shCTL, shLdha1, and shLdha2 groups (*n* = 6–8 biological replicates per group). Data are presented as the mean ± SEM. *P* values were calculated using the Kruskal–Wallis test (**B** and **E**). ***, *P* < 0.001 vs. shCTL.

To evaluate the impact of Ldha suppression on LM8 osteosarcoma cells *in vivo*, we subcutaneously implanted LM8-shCTL or -shLdha cells into six-week-old female C3H mice. *Ldha* knockdown in LM8 osteosarcoma cells had no effect on primary tumor growth in mice (Fig. 2A and B; Supplementary Fig. S1A). Notably, lung metastases were remarkably fewer in mice with LM8-shLdha tumors than in mice with shCTL tumors (Fig. 2C–E; Supplementary Fig. S1B).

### *Ldha* knockdown downregulates Hedgehog signaling

To elucidate how *Ldha* knockdown suppresses cell migration and invasion *in vitro* and reduces lung metastasis *in vivo*, we performed RNA-seq analysis. We examined Ldha-dependent changes in gene expression using LM8-shCTL and -shLdha cells.

**Figure 3.**
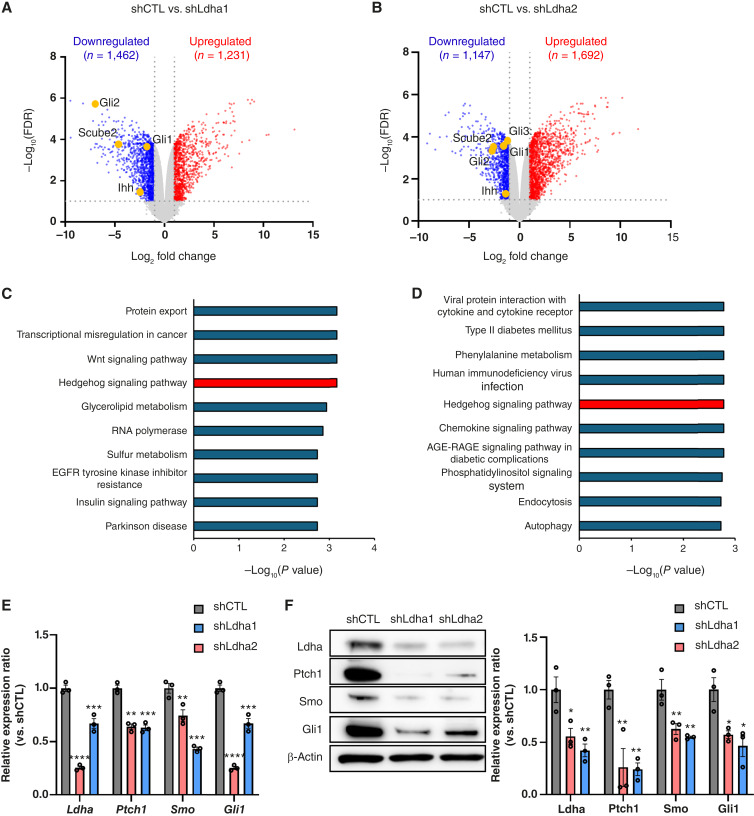
*LDHA* knockdown leads to the downregulation of Hedgehog signaling. **A,** Volcano plot illustrating false discovery rate and log_2_ fold change in gene expression between LM8-shCTL and shLdha1 cells. **B,** Volcano plot displaying false discovery rate and log_2_ fold change in gene expression between LM8-shCTL and shLdha2 cells. **C,** Kyoto Encyclopedia of Genes and Genomes (KEGG) pathway analysis of significantly upregulated and downregulated genes from (**A**; LM8-shLdha1 vs. LM8-shCTL). The top 10 significantly enriched terms are displayed. **D,** KEGG pathway analysis of significantly upregulated and downregulated genes from (**B**; LM8-shLdha2 vs. LM8-shCTL). The top 10 significantly enriched terms are displayed. **E,** mRNA expression levels of Hedgehog signaling–related genes (*n* = 3; independent experiments). The expression level of shCTL was set to 1. **F,** Protein expression of Hedgehog signaling–related proteins in LM8-shCTL, LM8-shLdha1, and LM8-shLdha2 cells. Data are presented as the mean ± SEM. *P* values were calculated using one-way ANOVA test (**E** and **F**). *, *P* < 0.05; **, *P* < 0.01; ***, *P* < 0.001 vs. shCTL; ****, *P* < 0.0001.

In LM8-shLdha1 and -shLdha2 cells, 1,231 and 1,692 genes were significantly upregulated and 1,462 and 1,147 genes were significantly downregulated, respectively, compared with LM8-shCTL cells (Fig. 3A and B). Kyoto Encyclopedia of Genes and Genomes pathway enrichment analysis of differentially expressed genes further identified Hedgehog signaling as a common feature among the top 10 significantly enriched pathways in both LM8-shLdha1 and -shLdha2 cells (Fig. 3C and D). To investigate the correlation between *Ldha* and Hedgehog signaling in LM8 osteosarcoma cells, we examined the expression of key pathway components. RT-qPCR analysis showed a significant reduction in the expression levels of *Ptch1*, *Smo*, and *Gli1* mRNA in LM8-shLdha cells compared with LM8-shCTL cells (Fig. 3E). RT-PCR analysis further confirmed the expression of Hedgehog ligands (*Shh*, *Ihh*, and *Dhh*), revealing that *Ihh* was expressed in LM8 osteosarcoma cells and was downregulated following *Ldha* knockdown (Supplementary Fig. S2A and S2B). Western blotting demonstrated a significant decrease in Pcth1, Smo, and Gli1 expression (Fig. 3F). We used IHC staining to confirm that Gli1 expression, an indicator of Hedgehog signaling pathway activation, was reduced in lung metastases in mice subcutaneously injected with LM8-shLdha cells (Supplementary Fig. S3). These findings suggest that *Ldha* knockdown leads to the downregulation of Hedgehog signaling in LM8 osteosarcoma cells.

### Inhibition of Hedgehog signaling suppresses osteosarcoma cell migration and invasion *in vitro*

**Figure 4.**
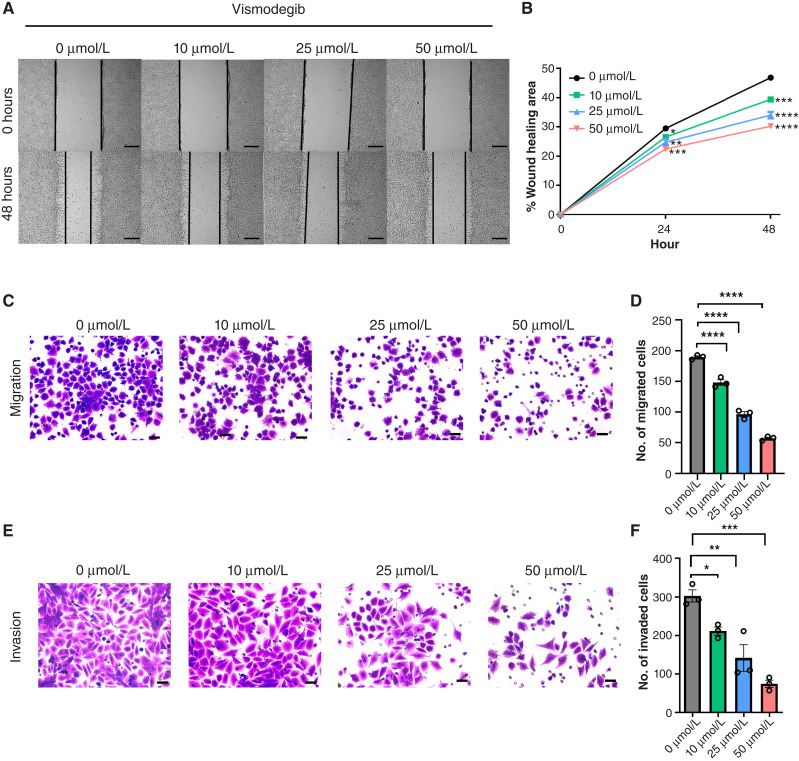
Vismodegib suppresses the proliferation, migration, and invasion of murine LM8 osteosarcoma cells *in vitro*. **A,** Wound healing assay to evaluate the migration capacity of vismodegib-treated LM8 cells. Scale bars, 200 μm. **B,** Quantification of wound healing rate from (**A**; *n* = 3; independent experiments). **C,** Transwell migration assay (without Matrigel) assessing migration capacity in vismodegib-treated LM8 cells. Scale bars, 50 μm. **D,** Quantification of migration ability from (**C**), showing the number of migrated cells after 24 hours (*n* = 3; independent experiments). **E,** Transwell invasion assay (with Matrigel) evaluating invasion capacity in vismodegib-treated LM8 cells. Scale bars, 50 μm. **F,** Quantification of invasion ability from (**E**), showing the number of invaded cells after 48 hours (*n* = 3; independent experiments). Data are presented as the mean ± SEM. *P* values were calculated using one-way ANOVA test (**B**, **D**, and **F**). *, *P* < 0.05; **, *P* < 0.01; ***, *P* < 0.001; and ****, *P* < 0.0001 vs. 0 μmol/L.

We examined the effects of Hedgehog signaling pathway inhibition on LM8 osteosarcoma cells. We used vismodegib (Smo inhibitor)—the first clinically approved Hedgehog pathway inhibitor that targets the most druggable member of the pathway. The CCK-8 assay showed that ≤50 μmol/L vismodegib had no effect on LM8 osteosarcoma cell proliferation (Supplementary Fig. S4). However, wound healing assays demonstrated that vismodegib significantly reduced cell migration (Fig. 4A and B). Transwell migration (Fig. 4C and D) and invasion assays (Fig. 4E and F) revealed that migrative and invasive abilities were suppressed in a concentration-dependent manner. We also confirmed that the migrative and invasive abilities of LM8-shLdha cells were restored by treatment with purmorphamine, an activator of Hedgehog signaling pathway (Supplementary Fig. S4B and S4C). These findings align with the results of *Lhda* knockdown in LM8 osteosarcoma cells, suggesting that Ldha regulates osteosarcoma cell migration and invasion through Hedgehog signaling.

### Inhibition of Hedgehog signaling reduces lung metastasis *in vivo*

**Figure 5.**
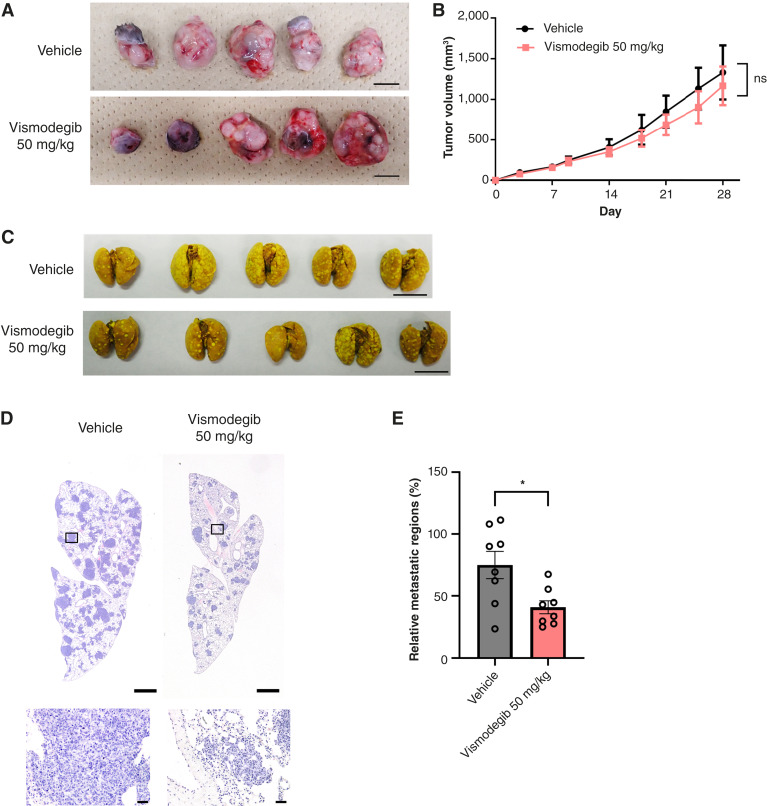
Vismodegib suppresses lung metastasis of murine osteosarcoma *in vivo*. **A,** Representative tumor images from vehicle (DMSO)- and vismodegib-treated mice subcutaneously implanted with LM8 cells. Scale bars, 10 mm. **B,** Tumor growth curves based on volume measurements at the indicated time points (*n* = 8 biological replicates per group). **C,** Bouin’s solution–fixed lung tissues from vehicle- and vismodegib-treated mice. Scale bars, 10 mm. **D,** Representative hematoxylin and eosin–stained lung sections from the vehicle- and vismodegib-treated groups. The top row at low magnification (scale bars, 1,000 μm) and the bottom at high magnification (scale bars, 50 μm). **E,** Quantification of metastatic area relative to total lung area in vehicle- and vismodegib-treated groups (*n* = 8 biological replicates per group). Data are presented as the mean ± SEM or median (IQR). *P* values were calculated using the Mann–Whitney *U* test (**B** and **E**). *, *P* < 0.05 vs. vehicle.

Mice were treated with vismodegib to determine whether inhibiting Hedgehog signaling affects primary tumor progression and lung metastasis *in vivo*. Vismodegib treatment did not cause weight loss or mortality in mice (Supplementary Fig. S5A and S5B). Although vismodegib did not inhibit primary tumor growth (Fig. 5A and B; Supplementary Fig. S5C), 50 mg/kg vismodegib significantly reduced lung metastasis compared with the vehicle control (Fig. 5C–E; Supplementary Fig. S5D). Furthermore, we confirmed that Gli1 expression was reduced in the vismodegib-treated group compared with the DMSO-treated group (Supplementary Fig. S5E). These findings indicate that Hedgehog signaling pathway inhibition suppresses LM8 osteosarcoma cell migration and invasion *in vitro* and reduces lung metastasis *in vivo*.

### *LDHA* knockdown downregulates Hedgehog signaling in human osteosarcoma cells

**Figure 6.**
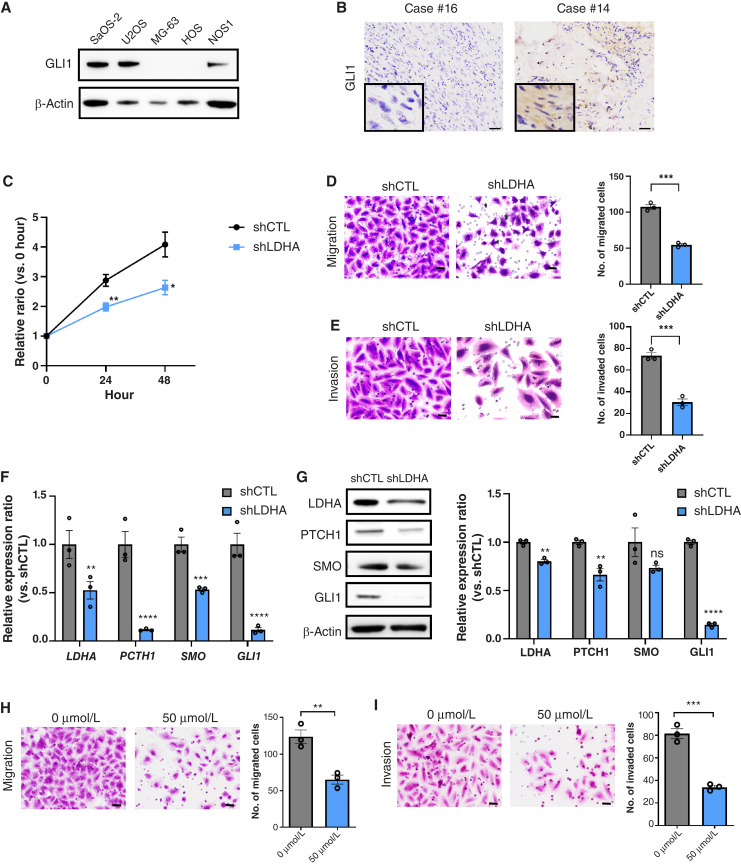
LDHA–Hedgehog signaling axis regulates the migration and invasion of human osteosarcoma cells. **A,** Protein expression of GLI1 in human osteosarcoma cell lines. **B,** IHC staining of human osteosarcoma specimens using an anti-GLI1 antibody. Representative cases with negative (left; case #16) and positive (right; case #14) GLI1 staining. Scale bars, 50 μm. **C,** Cell proliferation assay of SaOS-2-shCTL or shLDHA cells, assessed using CCK-8 (*n* = 8; independent experiments). The absorbance at 0 hour was set to 1. **D,** Transwell migration assay (without Matrigel) to assess migration capacity in SaOS-2-shCTL or shLDHA cells. Graph quantifying migration ability, showing the number of migrated cells after 24 hours (*n* = 3; independent experiments). Scale bars, 50 μm. **E,** Transwell invasion assay (without Matrigel) to assess migration capacity in SaOS-2-shCTL or shLDHA cells. Graph quantifying invasion ability, showing the number of invaded cells after 48 hours (*n* = 3; independent experiments). Scale bars, 50 μm. **F,** mRNA expression levels of *LDHA* mRNA and Hedgehog signaling–related genes in SaOS-2 cells with or without *LDHA* knockdown (*n* = 3; independent experiments). **G,** Protein expression levels of LDHA and Hedgehog signaling components in SaOS-2 cells with or without *LDHA* knockdown. Quantification of protein expression normalized to β-actin and represented in the bar graph (*n* = 3; independent experiments). **H,** Transwell migration assay (without Matrigel) evaluating the migration capacity of vismodegib-treated SaOS-2 cells. Graph quantifying migration ability, showing the number of migrated cells after 24 hours (*n* = 3; independent experiments). Scale bars, 50 μm. **I,** Transwell invasion assay (with Matrigel) assessing the invasion capacity of vismodegib-treated SaOS-2 cells. Graph quantifying invasion ability, showing the number of invaded cells after 48 hours (*n* = 3; independent experiments). Scale bars, 50 μm. Data are presented as the mean ± SEM. *P* values were calculated using an unpaired two-tailed Student *t* test (**C–I**). *, *P* < 0.05; **, *P* < 0.01; ***, *P* < 0.001; ****, *P* < 0.0001 vs. shCTL or 0 μmol/L.

Although *Ldha* knockdown and Hedgehog signaling inhibition suppressed migration and invasion in LM8 osteosarcoma cells *in vitro* and reduced lung metastasis *in vivo*, LM8 cells are of murine origin. Therefore, we investigated whether the LDHA–Hedgehog signaling axis regulates migration and invasion in human osteosarcoma cells. First, we examined GLI1 expression in human osteosarcoma cell lines and clinical samples. Western blotting detected GLI1 expression in three of five osteosarcoma cell lines: SaOS-2, U2OS, and NOS1 (Fig. 6A). IHC revealed GLI1-positive staining in 10 of 19 clinical specimens (Fig. 6B; Supplementary Fig. S6A and S6B), suggesting activation of Hedgehog signaling in ∼50% of all human osteosarcomas. Next, we assessed the role of LDHA in human osteosarcoma cells. *LDHA* knockdown through shRNA transfection suppressed the proliferation (Fig. 6C), migration (Fig. 6D), and invasion (Fig. 6E) of SaOS-2 cells. In addition, we investigated whether *LDHA* knockdown downregulates Hedgehog signaling. Similar to that in murine LM8 osteosarcoma cells, in SaOS-2 cells, *LDHA* knockdown through shRNA transfection decreased the expression of *PTCH1*, *SMO*, and *GLI1* mRNA (Fig. 6F) and decreased the levels of PTCH1 and GLI1 (Fig. 6G). We confirmed these findings in NOS1 cells. *LDHA* knockdown through siRNA transfection suppressed the expression of *PTCH1* and *GLI1* mRNA, cell migration, and invasion in NOS1 cells (Supplementary Fig. S7A and S7B). Vismodegib significantly impaired SaOS-2 and NOS1 cell migration and invasion in Transwell assays (Fig. 6H and I; Supplementary Fig. S7C). Overall, these findings indicate that the LDHA–Hedgehog signaling axis positively regulates migration and invasion in human osteosarcoma cells.

## Discussion

Hedgehog signaling, which is evolutionarily conserved across species from insects to mammals, plays a crucial role in embryonic and postnatal development by regulating cell proliferation, differentiation, tissue formation, homeostasis, and epithelial–mesenchymal transition (EMT; refs. 33–36). Dysregulation of Hedgehog signaling contributes to tumorigenesis and malignancy in various cancers, including basal cell carcinoma (37, 38); medulloblastoma (39); and lung, breast (40), pancreatic (41, 42), ovarian (43), prostate, and gastric cancers (44). Hedgehog signaling has been implicated in lung metastasis of prostate cancer (45) and liver metastasis of colorectal cancer (46). In osteosarcoma, high GLI2 expression, a key transcription factor in the Hedgehog pathway, has been associated with poor clinical outcomes (47, 48). *GLI2* knockdown enhances the sensitivity of osteosarcoma cells to chemotherapy (48). The inhibition of Hedgehog signaling has been found to reduce the expression of yes-associated protein 1—a potent oncogene that is highly expressed in both human and murine tumor tissues, thereby suppressing osteoblastic osteosarcoma progression (49). These findings suggest that Hedgehog signaling is involved in osteosarcoma tumorigenesis and malignancy.

In this study, we investigated the role of LDHA in osteosarcoma and found that *LDHA* knockdown reduced the migrative and invasive ability of murine osteosarcoma cells *in vitro* and decreased lung metastasis *in vivo*. RNA-seq analysis further revealed that *Ldha* knockdown downregulated Hedgehog signaling, and inhibition of Hedgehog signaling suppressed migration and invasion in murine osteosarcoma cells. Lung metastasis was significantly reduced in mice treated with a Hedgehog signaling pathway inhibitor. Similarly, in human osteosarcoma cells, *LDHA* knockdown led to the downregulation of Hedgehog signaling and a decrease in migrative and invasive ability. These findings suggest that the LDHA–Hedgehog signaling axis plays a role in the progression of lung metastasis in osteosarcoma.

Few studies have reported an association between LDHA and Hedgehog signaling in cancer. Han and colleagues (50) demonstrated that increased lactate production due to LDHA upregulation activated Hedgehog signaling and elevated SHH, SMO, and GLI1 expression in breast cancer. By contrast, we observed nonsignificant activation of Hedgehog signaling upon lactate addition in LM8 cells (Supplementary Fig. S8A). In addition, we could not confirm whether addition of lactate promoted cell migration and invasion in LM8 cells (Supplementary Fig. S8B and S8C). These findings suggest that in LM8 cells, an alternative molecular mechanism, rather than lactate-mediated regulation, regulates the interaction between LDHA and Hedgehog signaling.

Although Hedgehog signaling inhibition reduced lung metastases, the effect was less pronounced than that of *Ldha* silencing in LM8 cells. This finding suggests that Ldha promotes lung metastases in osteosarcoma through mechanisms in addition to the LDHA–Hedgehog signaling axis. Evidence indicates that LDHA contributes to tumor metastasis by regulating EMT-related proteins and ROS production, in addition to its role in lactate metabolism (51). First, studies have reported that LDHA facilitates tumor metastasis by inducing EMT in papillary thyroid carcinoma (23), oral squamous cell carcinoma (16), and pancreatic cancer (52). We evaluated the expression levels of EMT-related markers, such as *Cdh1*, *Cdh2*, *Vim*, and *Snai1*, although *Cdh1* and *Cdh2* were undetectable and the results for *Vim* and *Snai1* were inconsistent following *Ldha* knockdown in LM8 osteosarcoma cells (Supplementary Fig. S9), suggesting that EMT plays a minimal role in LDHA-driven osteosarcoma metastasis. Second, Arseneault and colleagues (53) reported that *LDHA* knockdown increased mitochondrial ROS levels, leading to altered protein disulfide bonding, which subsequently reduced cell proliferation and migration in melanoma cells. Similarly, *Ldha* knockdown in LM8 osteosarcoma cells showed a trend toward increased ROS levels (Supplementary Fig. S10A and S10B). Based on these findings, ROS could be involved in LDHA-mediated osteosarcoma metastasis.

This study has several limitations. First, although our findings suggest the involvement of an LDHA–Hedgehog axis in the regulation of lung metastasis in osteosarcoma, how LDHA suppression leads to the downregulation of Hedgehog pathway is unclear. In particular, direct causal evidence demonstrating that Hedgehog signaling mediates the antimetastatic effects of *LDHA* knockdown was not established.

Second, we used only one Hedgehog signal inhibitor in this study. Vismodegib is the first clinically approved Hedgehog pathway inhibitor that targets the most druggable member of the pathway (54). Baldanza and colleagues (55) reported that treatment with vismodegib suppresses proliferation and induces apoptosis in canine osteosarcoma cells. However, Nagao-Kitamoto and colleagues (47) showed that GANT61 (a GLI inhibitor) prevents migration and metastasis in human osteosarcoma cells in addition to vismodegib. Further experiments will be necessary to confirm the therapeutic potential of the LDHA–Hedgehog axis in osteosarcoma using other inhibitors.

We observed that local tumor size tended to be larger in the LM8-shLdha2 group than in the CTL group. By contrast, studies have reported that *LDHA* knockdown suppresses tumor growth in human osteosarcoma cells *in vivo* (24). These findings suggest differences in the phenotypic effects of *LDHA* knockdown between mouse- and human-derived osteosarcoma cells. However, because tumor engraftment was not achieved in the human osteosarcoma xenograft model in our study, we could not determine the effect of *LDHA* knockdown on local tumor progression in human osteosarcoma.

*LDHA* knockdown reduced GLI2 expression in both LM8 and SaOS-2 cells; however, the change was nonsignificant in NOS-1 cells (Supplementary Fig. S11). Although GLI2 has been implicated in osteosarcoma progression (47, 56), our data do not allow us to determine whether GLI2 functionally mediates the effects of LDHA suppression on metastasis. Further investigation will be required to clarify this correlation.

Finally, *in vivo* experiments were conducted using murine LM8 cells. However, validation in human-derived osteosarcoma models was not feasible in this study. Future studies employing clinically relevant human models will be important to confirm the translational relevance of these findings.

Despite these limitations, our findings provide evidence that LDHA suppression significantly inhibits lung metastasis in osteosarcoma. Our data supports a potential link between tumor metabolism and Hedgehog signaling in metastatic progression, positioning LDHA as a promising therapeutic target and providing a rationale for further mechanistic and translational investigations.

In conclusion, reducing LDHA expression suppressed lung metastasis in osteosarcoma, with Hedgehog signaling playing a role in this process. Our findings provide insights into the molecular mechanisms of osteosarcoma lung metastasis and suggest that the LDHA–Hedgehog signaling axis can be used as a therapeutic target for treating osteosarcoma lung metastasis.

## Supplementary Material

Supplementary Fig.1 Images of tumors and lung metastasis of all mice implanted with LM8-shCTL or shLdha cells

Supplementary Fig.2 mRNA expression of Hedgehog ligands in LM8 osteosarcoma cells

Supplementary Fig.3 Immunohistochemistry using an anti-GLI1 antibody in lung metastasis samples from mice bearing LM8-shCTL and shLdha cell-derived tumors

Supplementary Fig.4 Effects of vismodegib and purmorphamine on LM8 and LM8-shLdha osteosarcoma cells

Supplementary Fig.5 In vivo experiments

Supplementary Fig.6 Expression of Hedgehog signaling genes in the clinical samples and cells of human osteosarcoma

Supplementary Fig.7 LDHA knockdown downregulates Hedgehog signaling pathway in NOS1 osteosarcoma cells

Supplementary Fig.8 Effect of sodium lactate (NaLA) and lactic acid (LA) on Hedgehog signaling gene expression, migration, and invasion in LM8 osteosarcoma cells

Supplementary Fig.9 Expression levels of epithelial–mesenchymal transition-related genes in LM8-shCTL, shLdha1, and shLdha2 cells

Supplementary Fig.10 Ldha knockdown increases the generation of reactive oxygen species

Supplementary Fig.11 Expression levels of Gli2/GLI2 mRNA in LM8, SaOS-2, and NOS1 cells

Supplementary Fig.12 Full membrane images supporting Figs. 6A, 6G, and Supplementary Fig.6C 

Supplementary Table 1 Primer sequences used in this study

## Data Availability

The RNA-seq data have been submitted to the Gene Expression Omnibus database (RRID: SCR_005012) under the accession code GSE288144, and the uncropped blots are provided in Supplementary Fig. S12. All other data supporting the findings of this study are provided in the article and supplementary information files or are available from the corresponding author upon reasonable request.

## References

[1] Misaghi A , Goldin A, Awad M, Kulidjian AA. Osteosarcoma: a comprehensive review. SICOT J 2018;4:12. 29629690 10.1051/sicotj/2017028 PMC5890448

[2] Isakoff MS , Bielack SS, Meltzer P, Gorlick R. Osteosarcoma: current treatment and a collaborative pathway to success. J Clin Oncol 2015;33:3029–35. 26304877 10.1200/JCO.2014.59.4895 PMC4979196

[3] Bielack SS , Kempf-Bielack B, Delling G, Exner GU, Flege S, Helmke K, . Prognostic factors in high-grade osteosarcoma of the extremities or trunk: an analysis of 1,702 patients treated on neoadjuvant cooperative osteosarcoma study group protocols. J Clin Oncol 2002;20:776–90. 11821461 10.1200/JCO.2002.20.3.776

[4] Ganapathy-Kanniappan S , Geschwind JF. Tumor glycolysis as a target for cancer therapy: progress and prospects. Mol Cancer 2013;12:152. 24298908 10.1186/1476-4598-12-152 PMC4223729

[5] Sancho P , Barneda D, Heeschen C. Hallmarks of cancer stem cell metabolism. Br J Cancer 2016;114:1305–12. 27219018 10.1038/bjc.2016.152 PMC4984474

[6] Vander Heiden MG , Cantley LC, Thompson CB. Understanding the Warburg effect: the metabolic requirements of cell proliferation. Science 2009;324:1029–33. 19460998 10.1126/science.1160809 PMC2849637

[7] Pavlova NN , Zhu J, Thompson CB. The hallmarks of cancer metabolism: still emerging. Cell Metab 2022;34:355–77. 35123658 10.1016/j.cmet.2022.01.007 PMC8891094

[8] Pavlova NN , Thompson CB. The emerging hallmarks of cancer metabolism. Cell Metab 2016;23:27–47. 26771115 10.1016/j.cmet.2015.12.006 PMC4715268

[9] Hamanaka RB , Chandel NS. Cell biology. Warburg effect and redox balance. Science 2011;334:1219–20. 22144609 10.1126/science.1215637

[10] Ju HQ , Lin JF, Tian T, Xie D, Xu RH. NADPH homeostasis in cancer: functions, mechanisms and therapeutic implications. Signal Transduct Target Ther 2020;5:231. 33028807 10.1038/s41392-020-00326-0 PMC7542157

[11] Shi M , Cui J, Du J, Wei D, Jia Z, Zhang J, . A novel KLF4/LDHA signaling pathway regulates aerobic glycolysis in and progression of pancreatic cancer. Clin Cancer Res 2014;20:4370–80. 24947925 10.1158/1078-0432.CCR-14-0186 PMC4134726

[12] Cheng CS , Tan H, Wang N, Chen L, Meng Z, Chen Z, . Functional inhibition of lactate dehydrogenase suppresses pancreatic adenocarcinoma progression. Clin Transl Med 2021;11:e467. 34185423 10.1002/ctm2.467 PMC8238920

[13] Zhang W , Wang C, Hu X, Lian Y, Ding C, Ming L. Inhibition of LDHA suppresses cell proliferation and increases mitochondrial apoptosis via the JNK signaling pathway in cervical cancer cells. Oncol Rep 2022;47:77. 35191522 10.3892/or.2022.8288 PMC8892607

[14] He TL , Zhang YJ, Jiang H, Li XH, Zhu H, Zheng KL. The c-Myc-LDHA axis positively regulates aerobic glycolysis and promotes tumor progression in pancreatic cancer. Med Oncol 2015;32:187. 26021472 10.1007/s12032-015-0633-8 PMC4452209

[15] Wang ZY , Loo TY, Shen JG, Wang N, Wang DM, Yang DP, . LDH-A silencing suppresses breast cancer tumorigenicity through induction of oxidative stress mediated mitochondrial pathway apoptosis. Breast Cancer Res Treat 2012;131:791–800. 21452021 10.1007/s10549-011-1466-6

[16] Cai H , Li J, Zhang Y, Liao Y, Zhu Y, Wang C, . LDHA promotes oral squamous cell carcinoma progression through facilitating glycolysis and epithelial-mesenchymal transition. Front Oncol 2019;9:1446. 31921691 10.3389/fonc.2019.01446 PMC6930919

[17] Zhao Z , Han F, Yang S, Wu J, Zhan W. Oxamate-mediated inhibition of lactate dehydrogenase induces protective autophagy in gastric cancer cells: involvement of the Akt-mTOR signaling pathway. Cancer Lett 2015;358:17–26. 25524555 10.1016/j.canlet.2014.11.046

[18] Hirschhaeuser F , Sattler UG, Mueller-Klieser W. Lactate: a metabolic key player in cancer. Cancer Res 2011;71:6921–5. 22084445 10.1158/0008-5472.CAN-11-1457

[19] Wang JX , Choi SY, Niu X, Kang N, Xue H, Killam J, . Lactic acid and an acidic tumor microenvironment suppress anticancer immunity. Int J Mol Sci 2020;21:8363. 33171818 10.3390/ijms21218363 PMC7664620

[20] Yang Y , Chong Y, Chen M, Dai W, Zhou X, Ji Y, . Targeting lactate dehydrogenase a improves radiotherapy efficacy in non-small cell lung cancer: from bedside to bench. J Transl Med 2021;19:170. 33902615 10.1186/s12967-021-02825-2 PMC8074241

[21] Zhai X , Yang Y, Wan J, Zhu R, Wu Y. Inhibition of LDH-A by oxamate induces G2/M arrest, apoptosis and increases radiosensitivity in nasopharyngeal carcinoma cells. Oncol Rep 2013;30:2983–91. 24064966 10.3892/or.2013.2735

[22] Hua G , Liu Y, Li X, Xu P, Luo Y. Targeting glucose metabolism in chondrosarcoma cells enhances the sensitivity to doxorubicin through the inhibition of lactate dehydrogenase-A. Oncol Rep 2014;31:2727–34. 24789077 10.3892/or.2014.3156

[23] Hou X , Shi X, Zhang W, Li D, Hu L, Yang J, . LDHA induces EMT gene transcription and regulates autophagy to promote the metastasis and tumorigenesis of papillary thyroid carcinoma. Cell Death Dis 2021;12:347. 33795650 10.1038/s41419-021-03641-8 PMC8017009

[24] Gao S , Tu DN, Li H, Jiang JX, Cao X, You JB, . Pharmacological or genetic inhibition of LDHA reverses tumor progression of pediatric osteosarcoma. Biomed Pharmacother 2016;81:388–93. 27261617 10.1016/j.biopha.2016.04.029

[25] Chen H , Gao S, Cheng C. MiR-323a-3p suppressed the glycolysis of osteosarcoma via targeting LDHA. Hum Cell 2018;31:300–9. 30088225 10.1007/s13577-018-0215-0

[26] Li G , Li Y, Wang DY. Overexpression of miR-329-3p sensitizes osteosarcoma cells to cisplatin through suppression of glucose metabolism by targeting LDHA. Cell Biol Int 2021;45:766–74. 33058436 10.1002/cbin.11476

[27] Cui W , Lv W, Qu Y, Ma R, Wang YW, Xu YJ, . Discovery of 2-((3-cyanopyridin-2-yl)thio)acetamides as human lactate dehydrogenase A inhibitors to reduce the growth of MG-63 osteosarcoma cells: virtual screening and biological validation. Bioorg Med Chem Lett 2016;26:3984–7. 27406795 10.1016/j.bmcl.2016.06.083

[28] Dai H , Yi G, Jiang D, Min Y, Li Z. Circ_0000376 regulates miR-577/HK2/LDHA signaling pathway to promote the growth, invasion and glycolysis of osteosarcoma. J Orthop Surg Res 2024;19:67. 38218855 10.1186/s13018-023-04520-y PMC10788008

[29] Mei Z , Shen Z, Pu J, Liu Q, Liu G, He X, . NAT10 mediated ac4C acetylation driven m^6^A modification via involvement of YTHDC1-LDHA/PFKM regulates glycolysis and promotes osteosarcoma. Cell Commun Signal 2024;22:51. 38233839 10.1186/s12964-023-01321-y PMC10795323

[30] Jiang Y , Li F, Gao B, Ma M, Chen M, Wu Y, . KDM6B-mediated histone demethylation of LDHA promotes lung metastasis of osteosarcoma. Theranostics 2021;11:3868–81. 33664867 10.7150/thno.53347 PMC7914357

[31] Ge SX , Son EW, Yao R. iDEP: an integrated web application for differential expression and pathway analysis of RNA-Seq data. BMC Bioinformatics 2018;19:534. 30567491 10.1186/s12859-018-2486-6 PMC6299935

[32] Asai T , Ueda T, Itoh K, Yoshioka K, Aoki Y, Mori S, . Establishment and characterization of a murine osteosarcoma cell line (LM8) with high metastatic potential to the lung. Int J Cancer 1998;76:418–22. 9579581 10.1002/(sici)1097-0215(19980504)76:3<418::aid-ijc21>3.0.co;2-5

[33] Jing J , Wu Z, Wang J, Luo G, Lin H, Fan Y, . Hedgehog signaling in tissue homeostasis, cancers, and targeted therapies. Signal Transduct Target Ther 2023;8:315. 37596267 10.1038/s41392-023-01559-5 PMC10439210

[34] Skoda AM , Simovic D, Karin V, Kardum V, Vranic S, Serman L. The role of the Hedgehog signaling pathway in cancer: a comprehensive review. Bosn J Basic Med Sci 2018;18:8–20. 29274272 10.17305/bjbms.2018.2756 PMC5826678

[35] Pak E , Segal RA. Hedgehog signal transduction: key players, oncogenic drivers, and cancer therapy. Dev Cell 2016;38:333–44. 27554855 10.1016/j.devcel.2016.07.026 PMC5017307

[36] Ingham PW , McMahon AP. Hedgehog signaling in animal development: paradigms and principles. Genes Dev 2001;15:3059–87. 11731473 10.1101/gad.938601

[37] Xie J , Murone M, Luoh SM, Ryan A, Gu Q, Zhang C, . Activating Smoothened mutations in sporadic basal-cell carcinoma. Nature 1998;391:90–2. 9422511 10.1038/34201

[38] Basset-Seguin N , Sharpe HJ, de Sauvage FJ. Efficacy of Hedgehog pathway inhibitors in basal cell carcinoma. Mol Cancer Ther 2015;14:633–41. 25585509 10.1158/1535-7163.MCT-14-0703

[39] Di Magno L , Manzi D, D’Amico D, Coni S, Macone A, Infante P, . Druggable glycolytic requirement for Hedgehog-dependent neuronal and medulloblastoma growth. Cell Cycle 2014;13:3404–13. 25485584 10.4161/15384101.2014.952973 PMC4613849

[40] Benvenuto M , Masuelli L, De Smaele E, Fantini M, Mattera R, Cucchi D, . In vitro and in vivo inhibition of breast cancer cell growth by targeting the Hedgehog/GLI pathway with SMO (GDC-0449) or GLI (GANT-61) inhibitors. Oncotarget 2016;7:9250–70. 26843616 10.18632/oncotarget.7062 PMC4891038

[41] Bailey JM , Mohr AM, Hollingsworth MA. Sonic hedgehog paracrine signaling regulates metastasis and lymphangiogenesis in pancreatic cancer. Oncogene 2009;28:3513–25. 19633682 10.1038/onc.2009.220 PMC2910592

[42] Singh BN , Fu J, Srivastava RK, Shankar S. Hedgehog signaling antagonist GDC-0449 (Vismodegib) inhibits pancreatic cancer stem cell characteristics: molecular mechanisms. PLoS One 2011;6:e27306. 22087285 10.1371/journal.pone.0027306 PMC3210776

[43] Pan Y , Zhou J, Zhang W, Yan L, Lu M, Dai Y, . The Sonic Hedgehog signaling pathway regulates autophagy and migration in ovarian cancer. Cancer Med 2021;10:4510–21. 34076346 10.1002/cam4.4018 PMC8267163

[44] Ke B , Wang XN, Liu N, Li B, Wang XJ, Zhang RP, . Sonic Hedgehog/Gli1 signaling pathway regulates cell migration and invasion via induction of epithelial-to-mesenchymal transition in gastric cancer. J Cancer 2020;11:3932–43. 32328197 10.7150/jca.42900 PMC7171499

[45] Karhadkar SS , Bova GS, Abdallah N, Dhara S, Gardner D, Maitra A, . Hedgehog signalling in prostate regeneration, neoplasia and metastasis. Nature 2004;431:707–12. 15361885 10.1038/nature02962

[46] Tan Y , Hu Y, Xiao Q, Tang Y, Chen H, He J, . Silencing of brain-expressed X-linked 2 (BEX2) promotes colorectal cancer metastasis through the Hedgehog signaling pathway. Int J Biol Sci 2020;16:228–38. 31929751 10.7150/ijbs.38431 PMC6949152

[47] Nagao-Kitamoto H , Nagata M, Nagano S, Kitamoto S, Ishidou Y, Yamamoto T, . GLI2 is a novel therapeutic target for metastasis of osteosarcoma. Int J Cancer 2015;136:1276–84. 25082385 10.1002/ijc.29107

[48] Yang W , Liu X, Choy E, Mankin H, Hornicek FJ, Duan Z. Targeting hedgehog-GLI-2 pathway in osteosarcoma. J Orthop Res 2013;31:502–9. 22968906 10.1002/jor.22230

[49] Chan LH , Wang W, Yeung W, Deng Y, Yuan P, Mak KK. Hedgehog signaling induces osteosarcoma development through Yap1 and H19 overexpression. Oncogene 2014;33:4857–66. 24141783 10.1038/onc.2013.433

[50] Han J , Chen X, Wang J, Liu B. Glycolysis-related lncRNA TMEM105 upregulates LDHA to facilitate breast cancer liver metastasis via sponging miR-1208. Cell Death Dis 2023;14:80. 36737428 10.1038/s41419-023-05628-z PMC9898275

[51] Feng Y , Xiong Y, Qiao T, Li X, Jia L, Han Y. Lactate dehydrogenase A: a key player in carcinogenesis and potential target in cancer therapy. Cancer Med 2018;7:6124–36. 30403008 10.1002/cam4.1820 PMC6308051

[52] Wu C , Zheng C, Chen S, He Z, Hua H, Sun C, . FOXQ1 promotes pancreatic cancer cell proliferation, tumor stemness, invasion and metastasis through regulation of LDHA-mediated aerobic glycolysis. Cell Death Dis 2023;14:699. 37875474 10.1038/s41419-023-06207-y PMC10598070

[53] Arseneault R , Chien A, Newington JT, Rappon T, Harris R, Cumming RC. Attenuation of LDHA expression in cancer cells leads to redox-dependent alterations in cytoskeletal structure and cell migration. Cancer Lett 2013;338:255–66. 23583676 10.1016/j.canlet.2013.03.034

[54] Kumar RM , Fuchs B. Hedgehog signaling inhibitors as anti-cancer agents in osteosarcoma. Cancers (Basel) 2015;7:784–94. 25985215 10.3390/cancers7020784 PMC4491684

[55] Baldanza VE , Rogic A, Yan W, Levine CB, Levine RA, Miller AD, . Evaluation of canonical Hedgehog signaling pathway inhibition in canine osteosarcoma. PLoS One 2020;15:e0231762. 32348319 10.1371/journal.pone.0231762 PMC7190150

[56] Nagao-Kitamoto H , Setoguchi T, Kitamoto S, Nakamura S, Tsuru A, Nagata M, . Ribosomal protein S3 regulates GLI2-mediated osteosarcoma invasion. Cancer Lett 2015;356:855–61. 25449781 10.1016/j.canlet.2014.10.042

