# Supplementary figures and images for: Ldha Regulates Osteosarcoma Lung Metastasis through Hedgehog Signaling
Source: Cancer Res Commun. 2026 Jun 25;6(6):1495–508. doi: 10.1158/2767-9764.CRC-25-0163 (PMC13295448; doi:10.1158/2767-9764.CRC-25-0163)

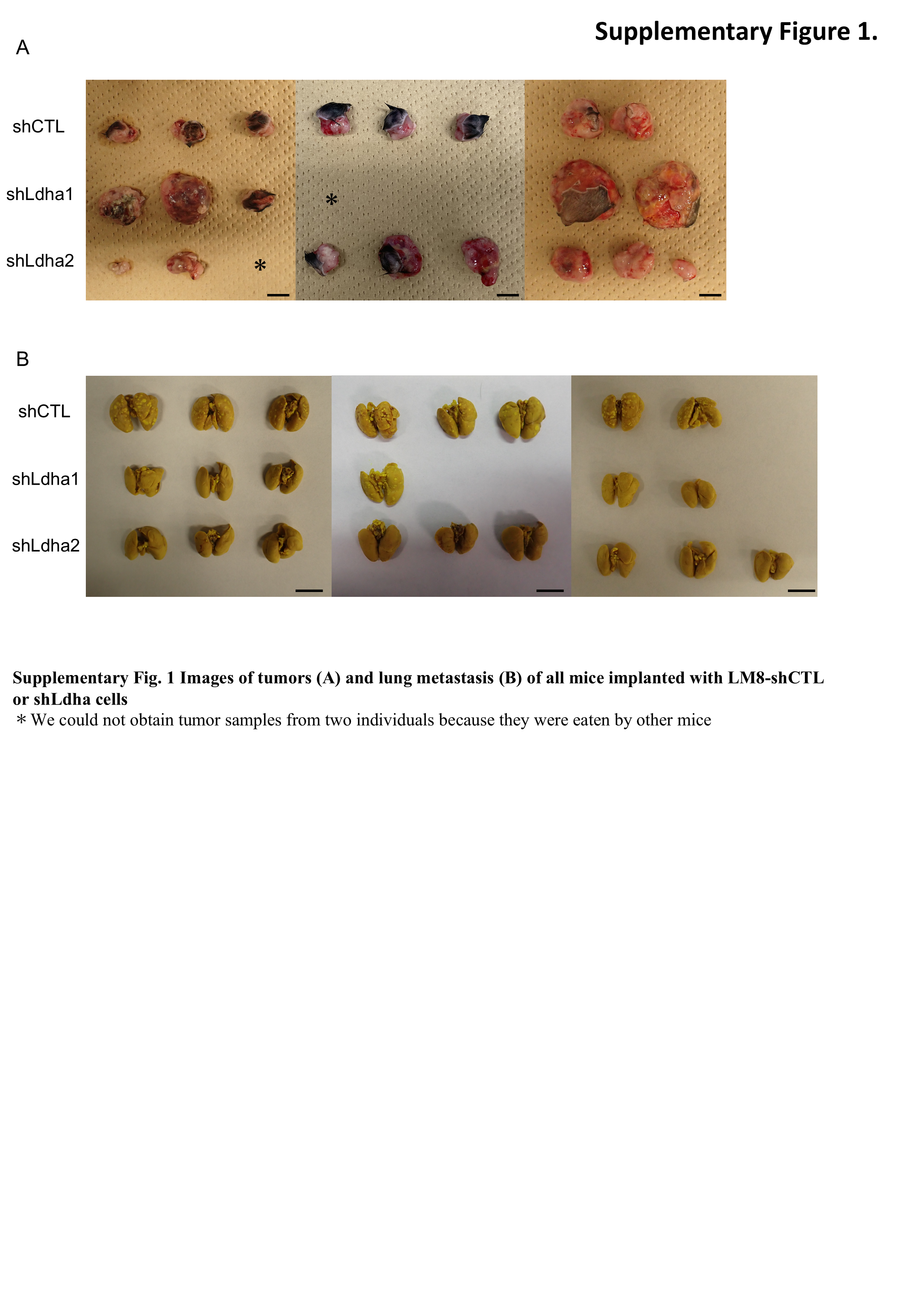

Supplement: Supplementary Fig.1 — Images of tumors and lung metastasis of all mice implanted with LM8-shCTL or shLdha cells [file crc-25-0163_supplementary_fig.1_suppsf1.png]

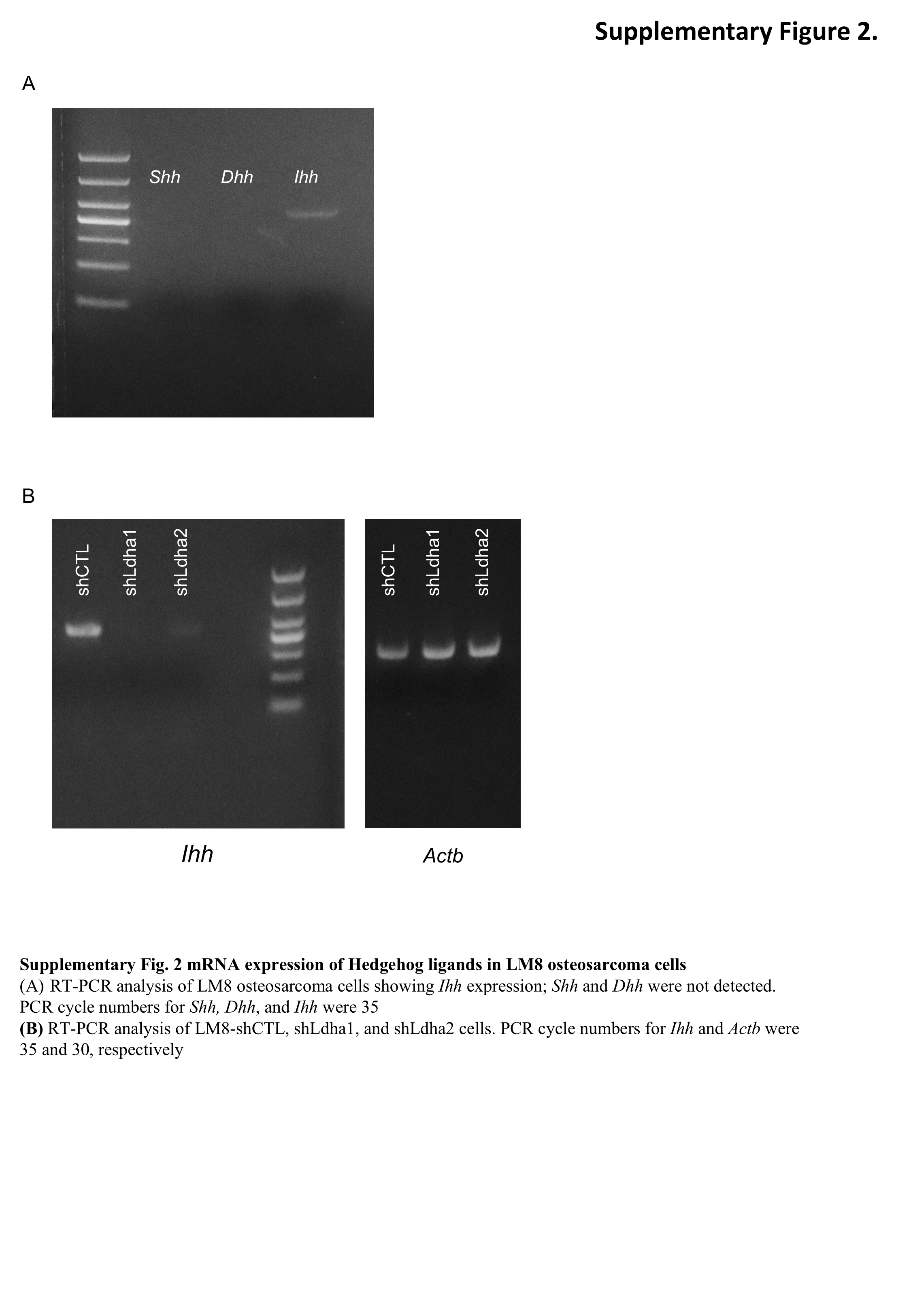

Supplement: Supplementary Fig.2 — mRNA expression of Hedgehog ligands in LM8 osteosarcoma cells [file crc-25-0163_supplementary_fig.2_suppsf2.png]

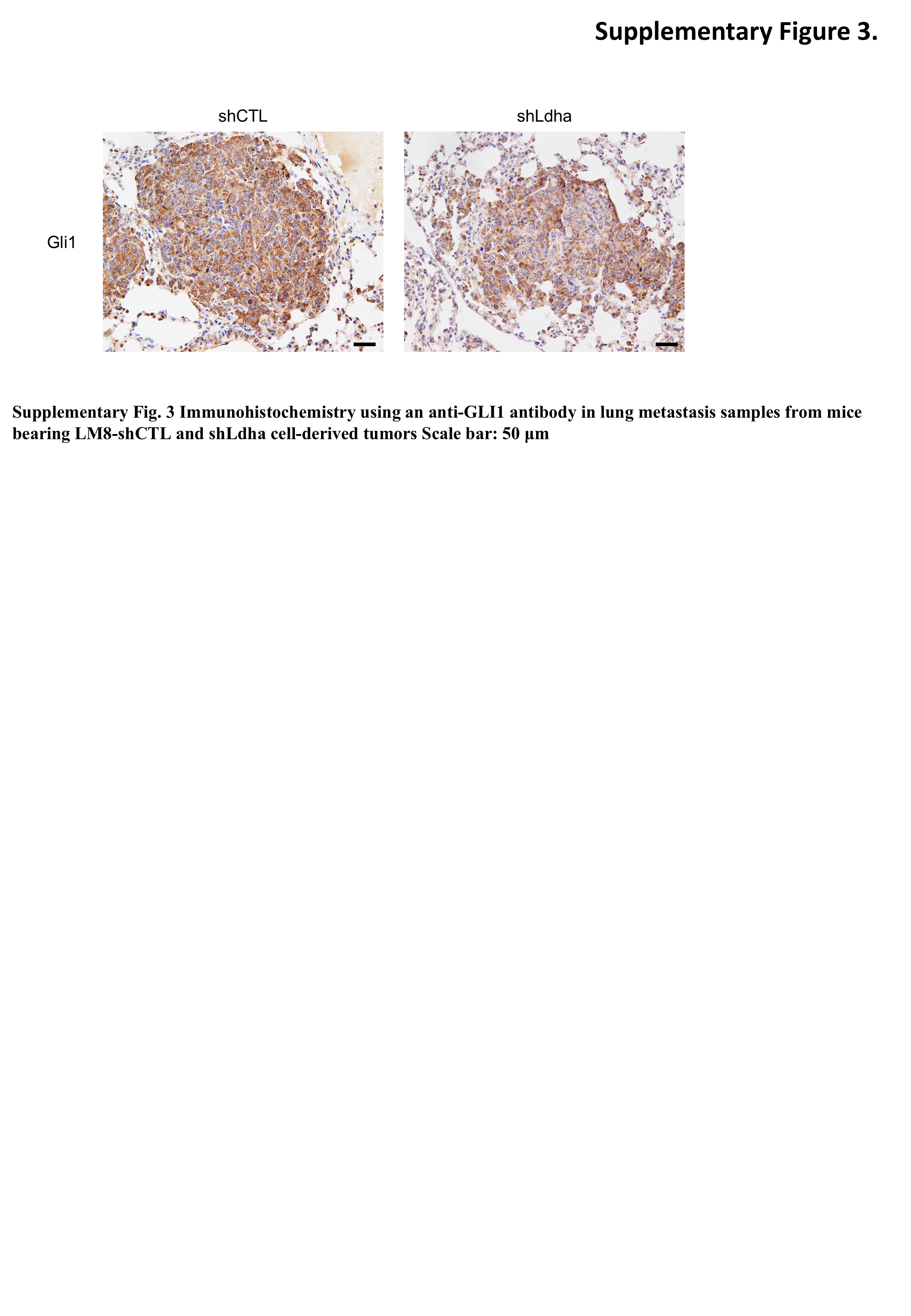

Supplement: Supplementary Fig.3 — Immunohistochemistry using an anti-GLI1 antibody in lung metastasis samples from mice bearing LM8-shCTL and shLdha cell-derived tumors [file crc-25-0163_supplementary_fig.3_suppsf3.png]

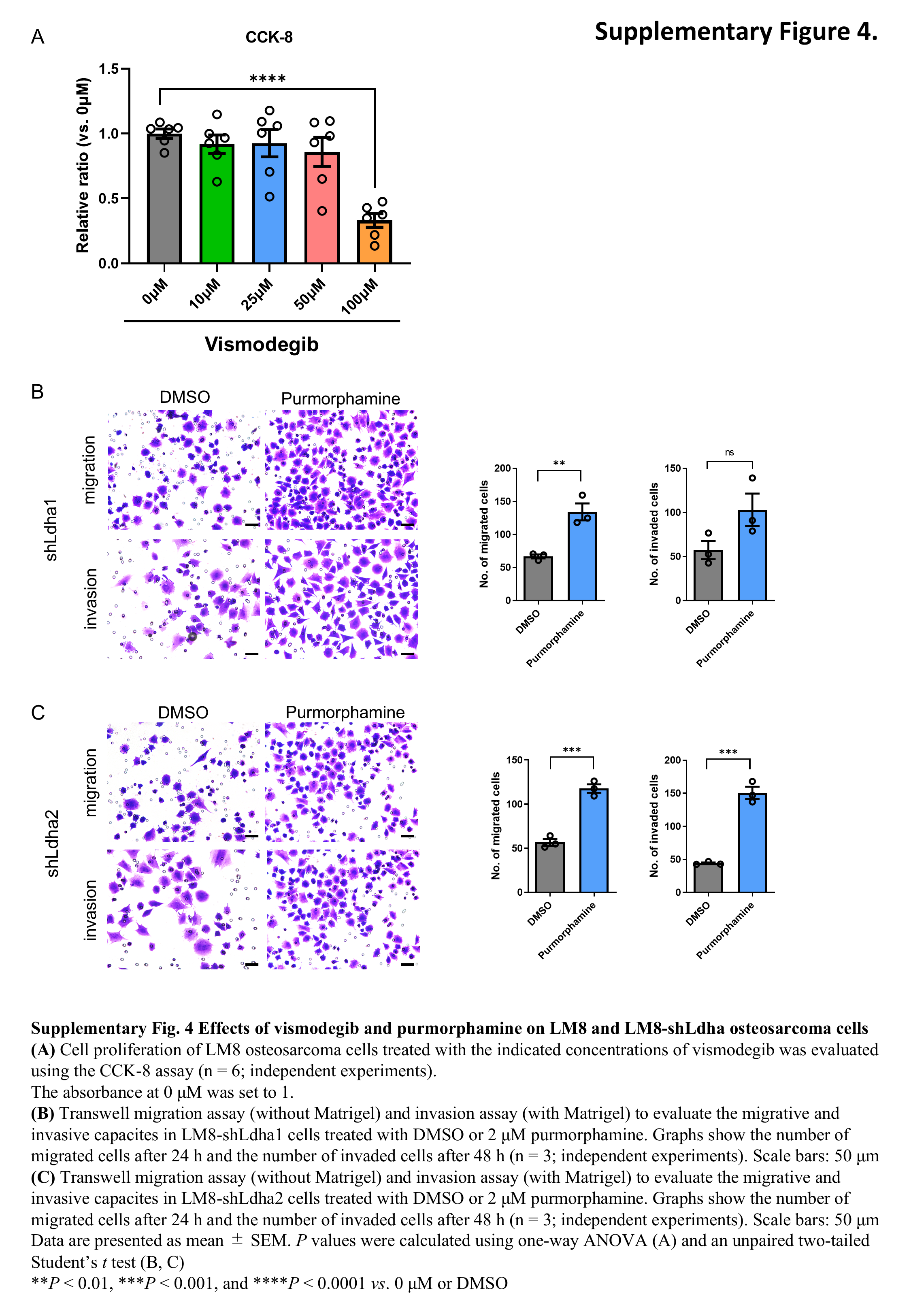

Supplement: Supplementary Fig.4 — Effects of vismodegib and purmorphamine on LM8 and LM8-shLdha osteosarcoma cells [file crc-25-0163_supplementary_fig.4_suppsf4.png]

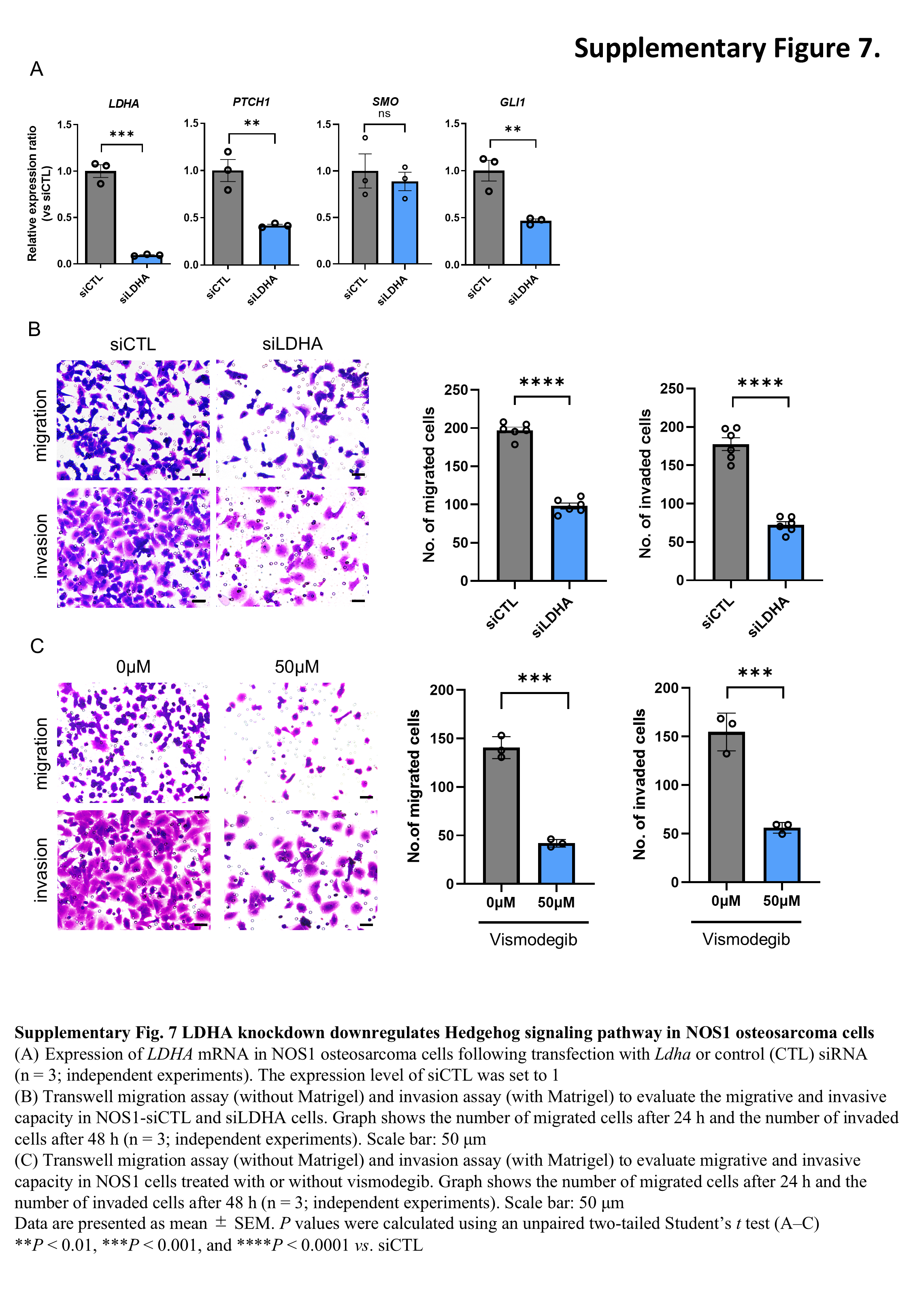

Supplement: Supplementary Fig.7 — LDHA knockdown downregulates Hedgehog signaling pathway in NOS1 osteosarcoma cells [file crc-25-0163_supplementary_fig.7_suppsf7.png]

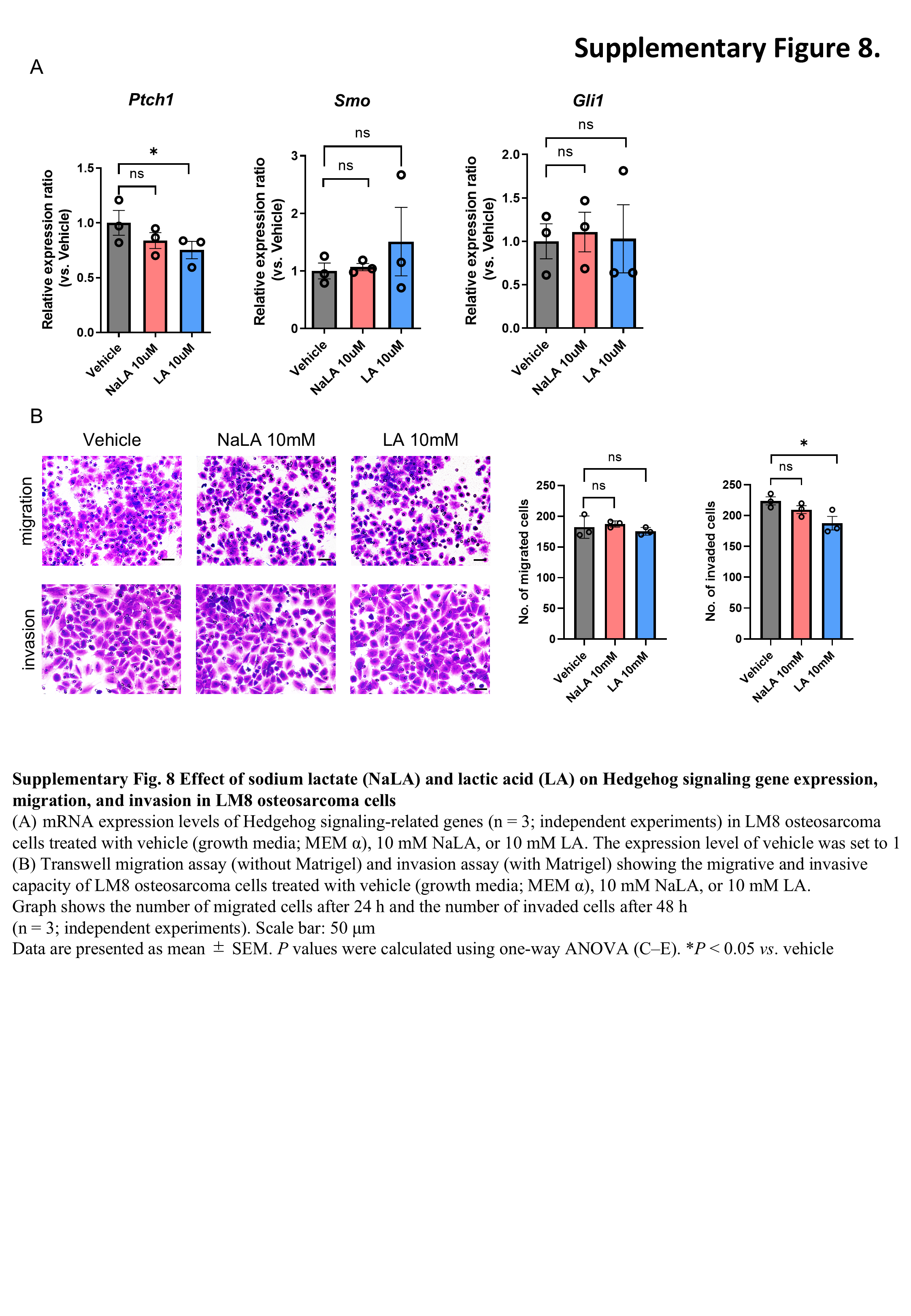

Supplement: Supplementary Fig.8 — Effect of sodium lactate (NaLA) and lactic acid (LA) on Hedgehog signaling gene expression, migration, and invasion in LM8 osteosarcoma cells [file crc-25-0163_supplementary_fig.8_suppsf8.png]

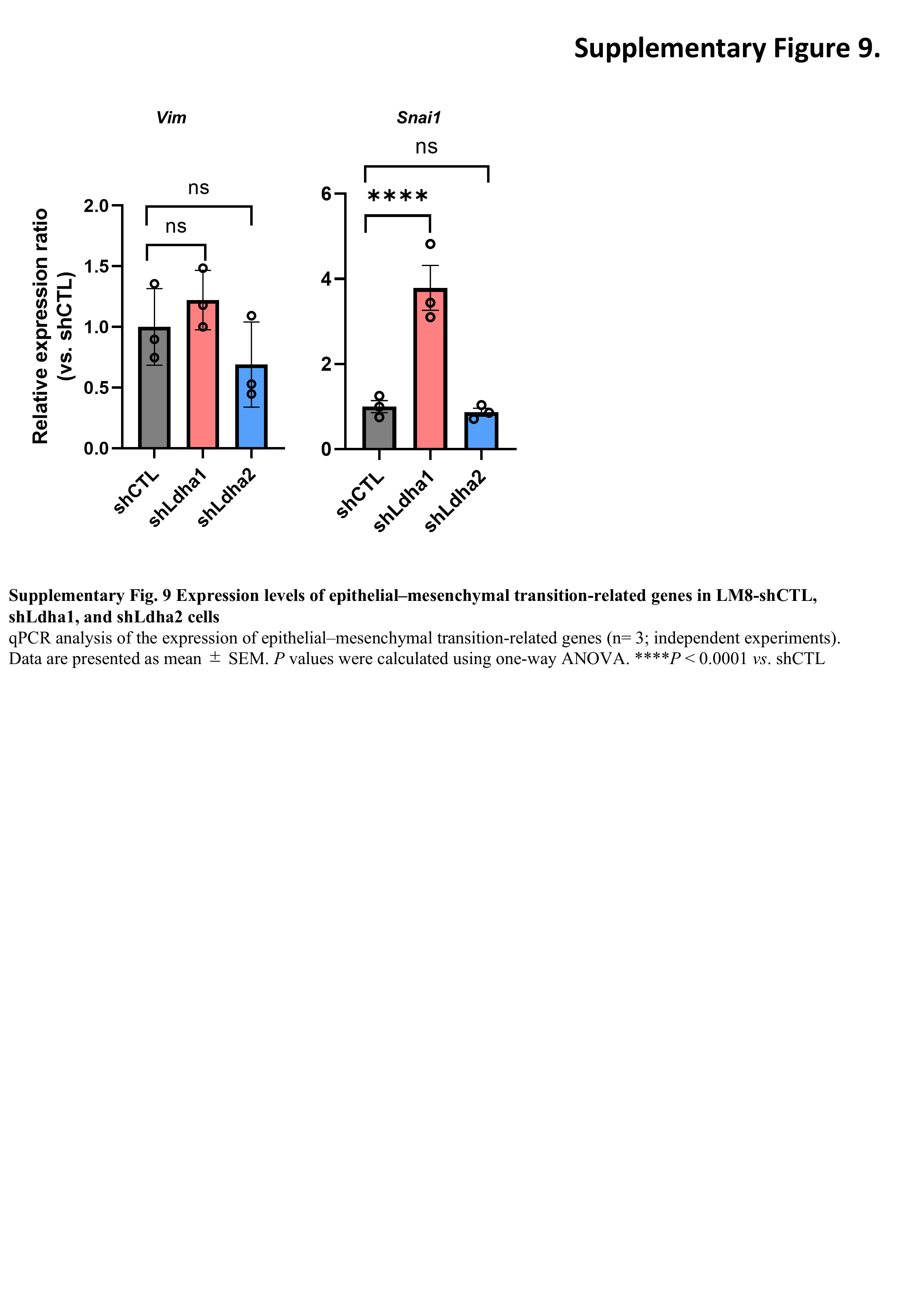

Supplement: Supplementary Fig.9 — Expression levels of epithelial–mesenchymal transition-related genes in LM8-shCTL, shLdha1, and shLdha2 cells [file crc-25-0163_supplementary_fig.9_suppsf9.png]

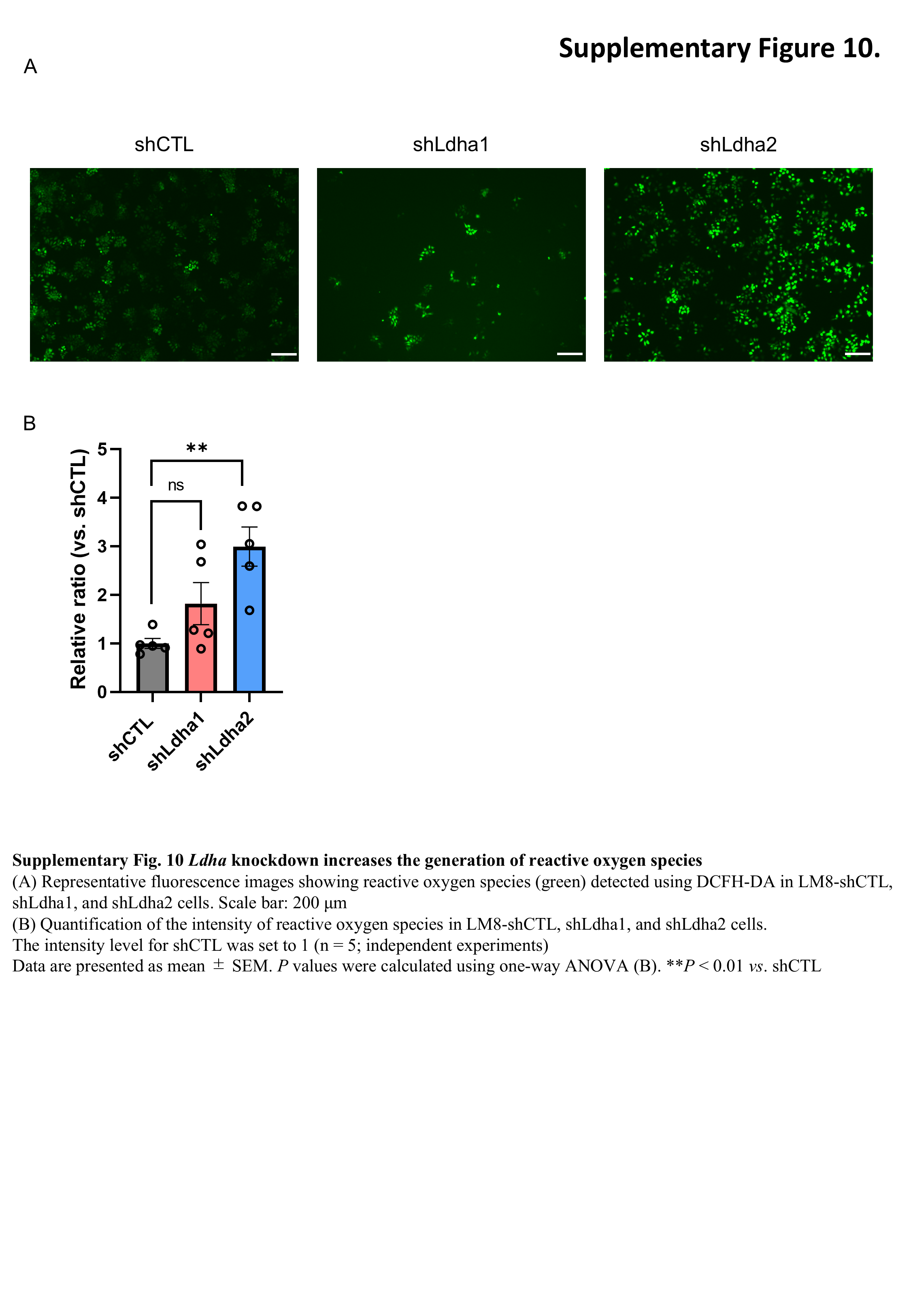

Supplement: Supplementary Fig.10 — Ldha knockdown increases the generation of reactive oxygen species [file crc-25-0163_supplementary_fig.10_suppsf10.png]

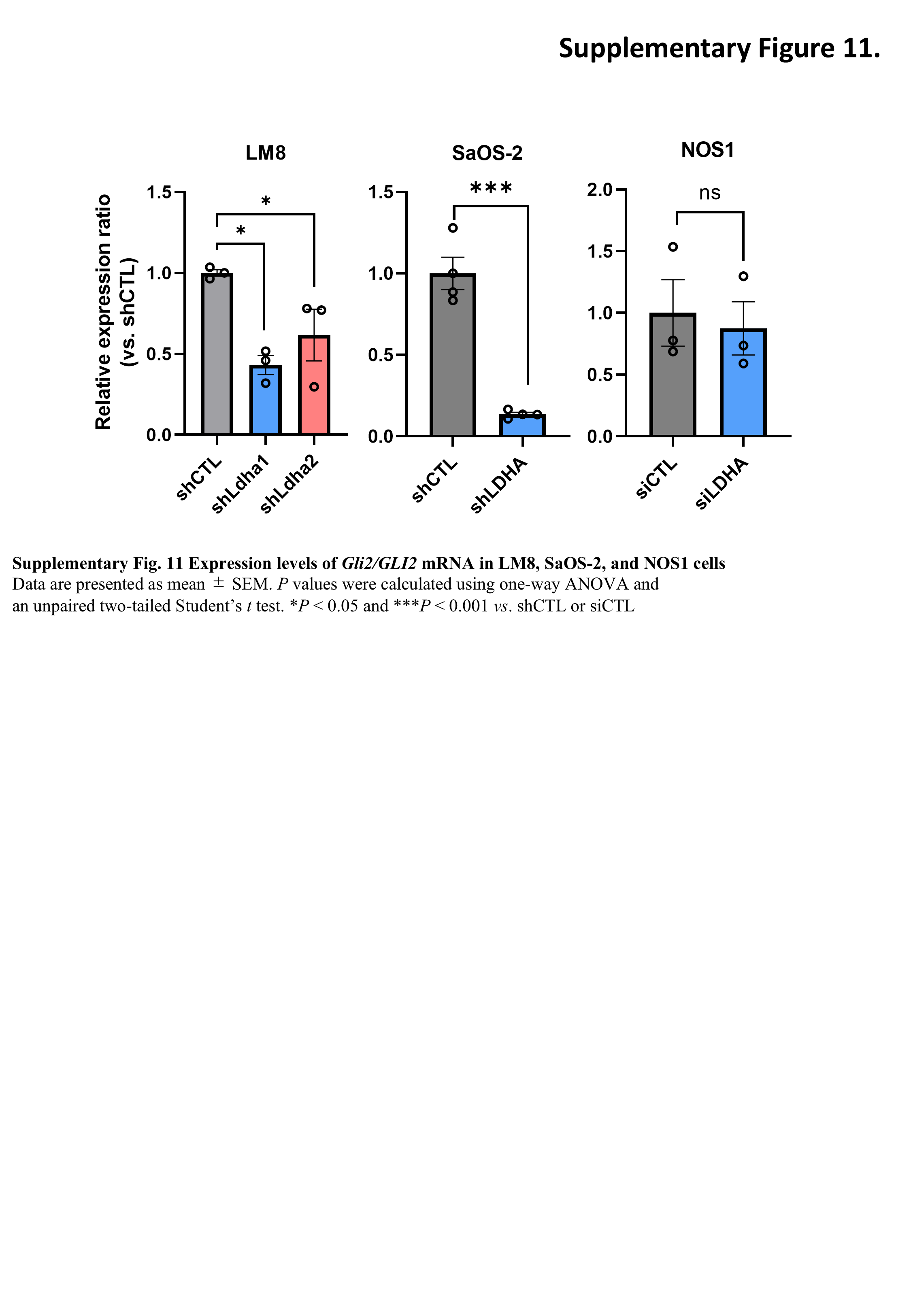

Supplement: Supplementary Fig.11 — Expression levels of Gli2/GLI2 mRNA in LM8, SaOS-2, and NOS1 cells [file crc-25-0163_supplementary_fig.11_suppsf11.png]

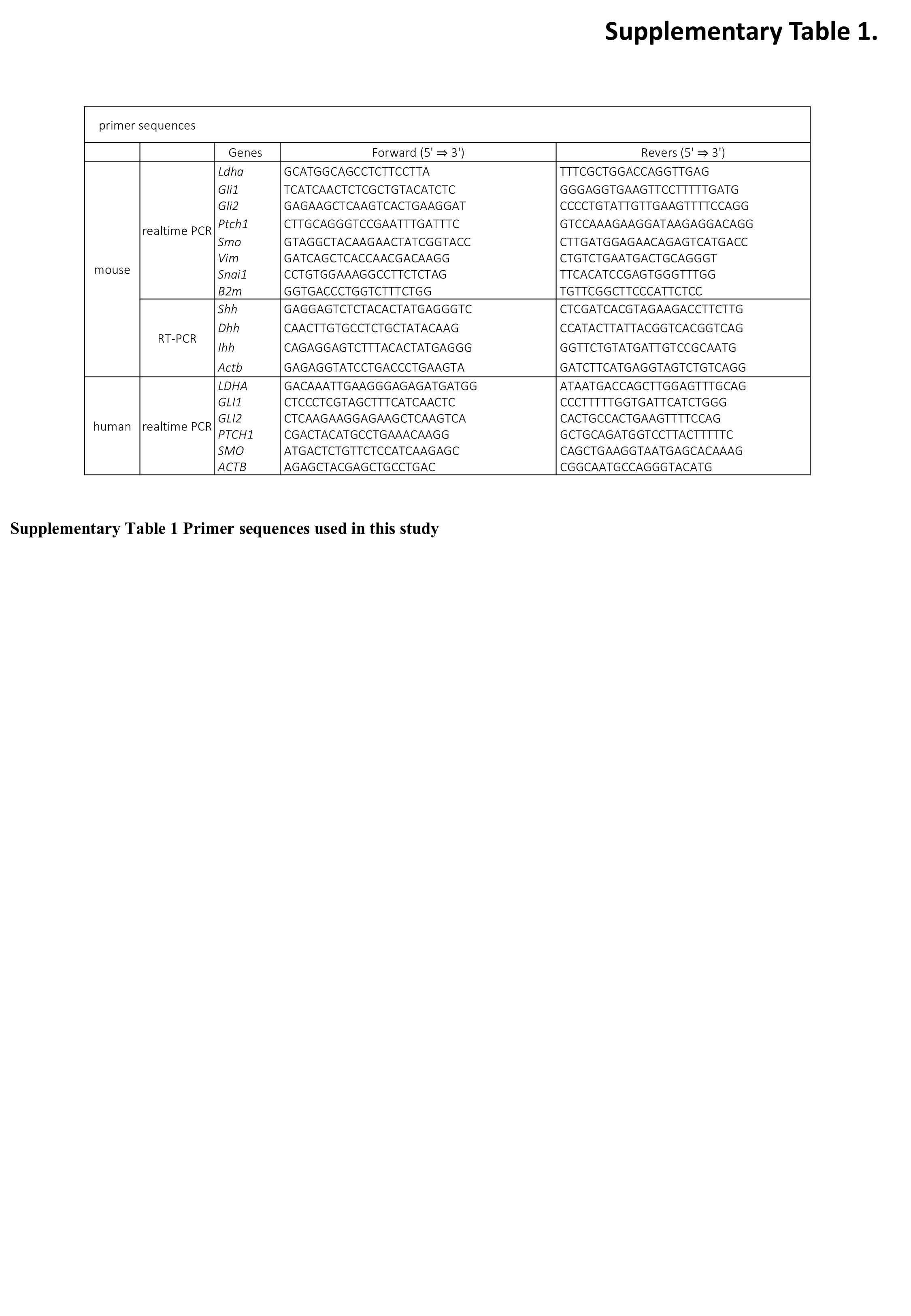

Supplement: Supplementary Table 1 — Primer sequences used in this study [file crc-25-0163_supplementary_table_1_suppst1.png]
