# Supplementary material for: Ldha Regulates Osteosarcoma Lung Metastasis through Hedgehog Signaling
Source: Cancer Res Commun. 2026 Jun 25;6(6):1495–508. doi: 10.1158/2767-9764.CRC-25-0163 (PMC13295448; doi:10.1158/2767-9764.CRC-25-0163)
Supplement: Supplementary Fig.5 — In vivo experiments [file crc-25-0163_supplementary_fig.5_suppsf5.pdf]

Supplementary Figure 5.

A

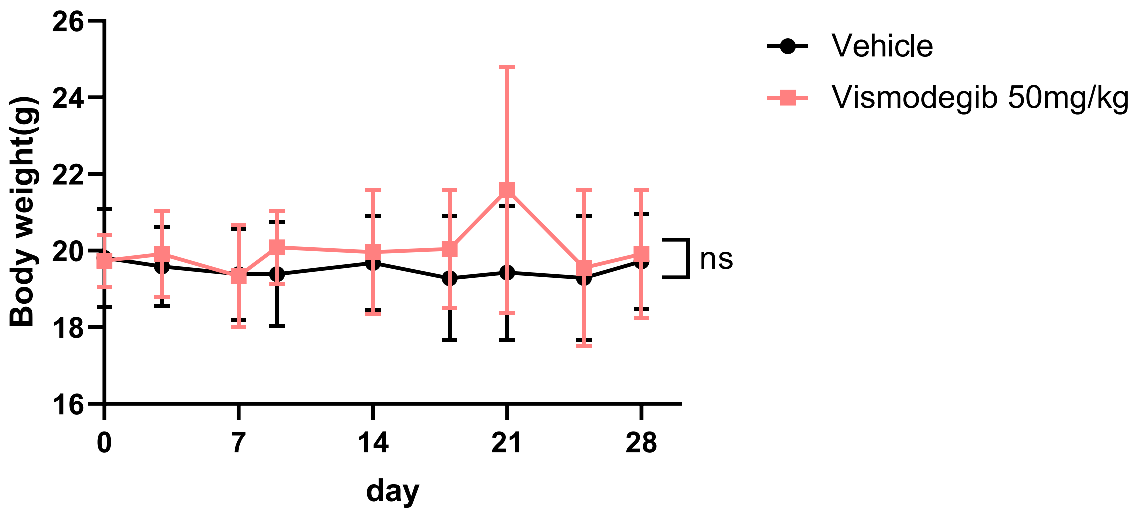

B

Vehicle								
	1	2	3	4	5	6	7	8
Day0	17.3	19.2	19.9	20.6	21.8	20.2	19.8	19.7
Day3	18	19.6	19.7	20.9	21.2	19.2	19.2	18.9
Day7	19.5	19.7	20	21.1	20.5	17.8	18	18.5
Day9	18.2	18.7	19.2	20.1	22.4	18.4	18.8	19.3
Day14	18.8	18.9	19.4	20.1	22.4	19.5	18.4	19.9
Day18	18.6	18.7	19.8	20.2	22.6	17.3	18.1	18.9
Day21	19.2	19.5	19.1	20.4	23.1	17.5	17.7	18.9
Day25	19.4	*N/A	20.3	21.1	21	16.8	18.4	18
Day28	20.6	21.3	20.9	20.6	19	18.1	18.7	18.6

Vismodegib 50mg/kg								
	1	2	3	4	5	6	7	8
Day0	20.4	19.3	19.2	19.8	20.6	20.3	18.6	19.7
Day3	22.1	19.8	20.8	19.2	19.6	20	18.3	19.5
Day7	21.4	19.9	20.3	18	19.4	20	17.4	18.3
Day9	21.6	19.3	19.4	19.8	20.7	20.6	18.7	20.6
Day14	22.7	17.1	19.8	19.7	20.3	20.8	18.8	20.5
Day18	22	18	20	*N/A	20.9	20.7	17.9	20.8
Day21	22	28.9	20.9	18.3	21.6	20.8	19	21.2
Day25	21.8	19	21.2	15.3	21.1	19.8	18.7	19.5
Day28	20.5	19.5	22.3	16.6	20.8	19.9	19	20.7

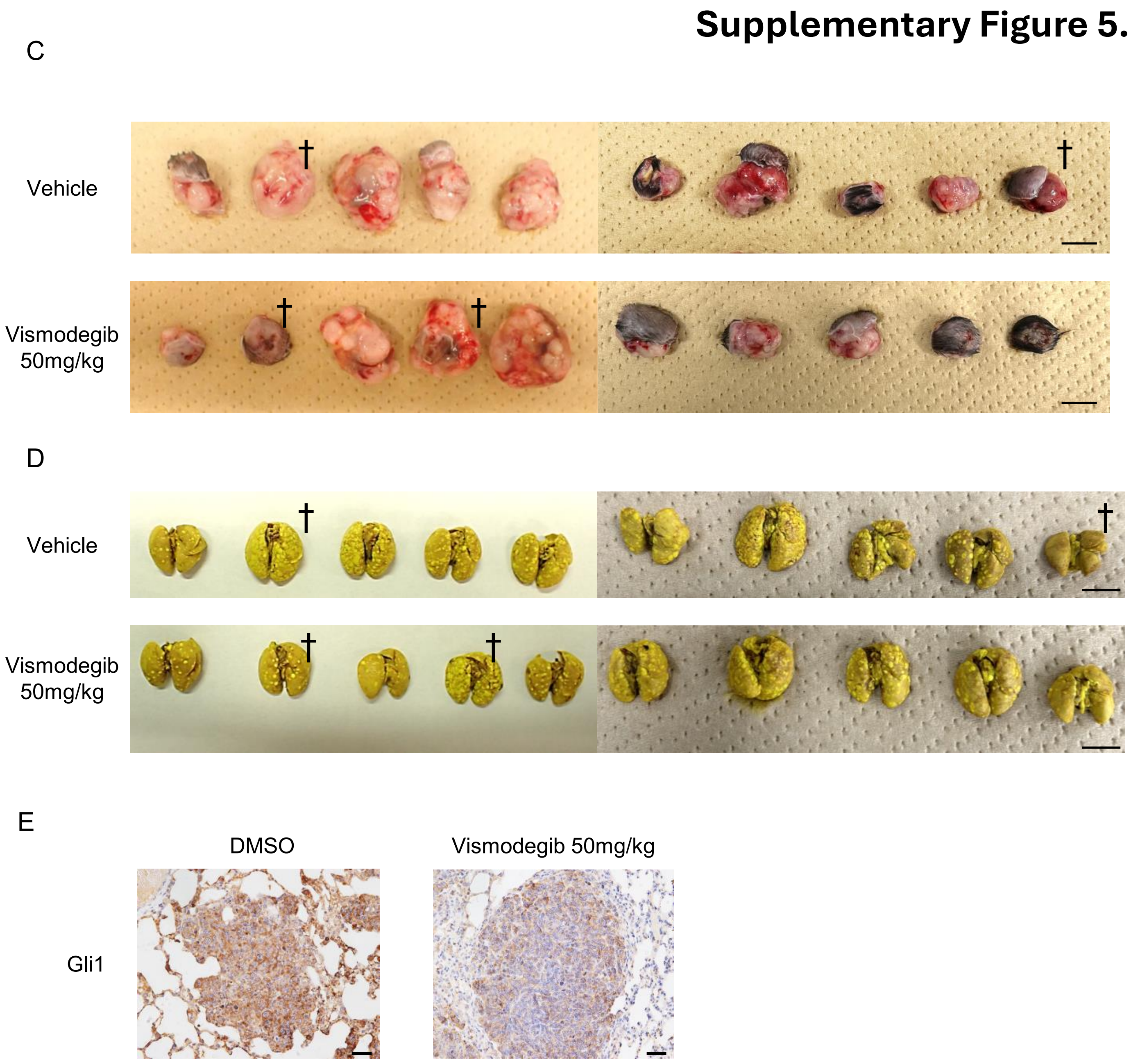

Supplementary Fig. 5 *In vivo* experiments
(A) Growth curves were generated by monitoring body weight (n = 8 biological replicates per group) at the indicated time points
(B) Tables show the body weight of individual mice at the indicated time points (n = 8 biological replicates per group).
*N/A denotes not applicable due to loss of data
(C) Images of tumors of individual mice from vehicle (DMSO)- and vismodegib-treated mice subcutaneously implanted with LM8 cells. Scale bar: 10 mm.
†Four individuals were excluded from further analysis to remove the highest and lowest outliers
(D) Images of lung metastasis of individual mice from vehicle (DMSO)- and vismodegib-treated mice subcutaneously implanted with LM8 cells. Scale bar: 10 mm.
†Four individuals were excluded from further analysis to remove the highest and lowest outliers
(E) Immunohistochemistry staining with anti-GLI1 antibody in lung metastasis samples treated with or without vismodegib.
Data are presented as mean ± SEM. *P* values were calculated using the Mann–Whitney *U* test (A)
