# Supplementary material for: Ldha Regulates Osteosarcoma Lung Metastasis through Hedgehog Signaling
Source: Cancer Res Commun. 2026 Jun 25;6(6):1495–508. doi: 10.1158/2767-9764.CRC-25-0163 (PMC13295448; doi:10.1158/2767-9764.CRC-25-0163)
Supplement: Supplementary Fig.6 — Expression of Hedgehog signaling genes in the clinical samples and cells of human osteosarcoma [file crc-25-0163_supplementary_fig.6_suppsf6.pdf]

Supplementary Figure 6.

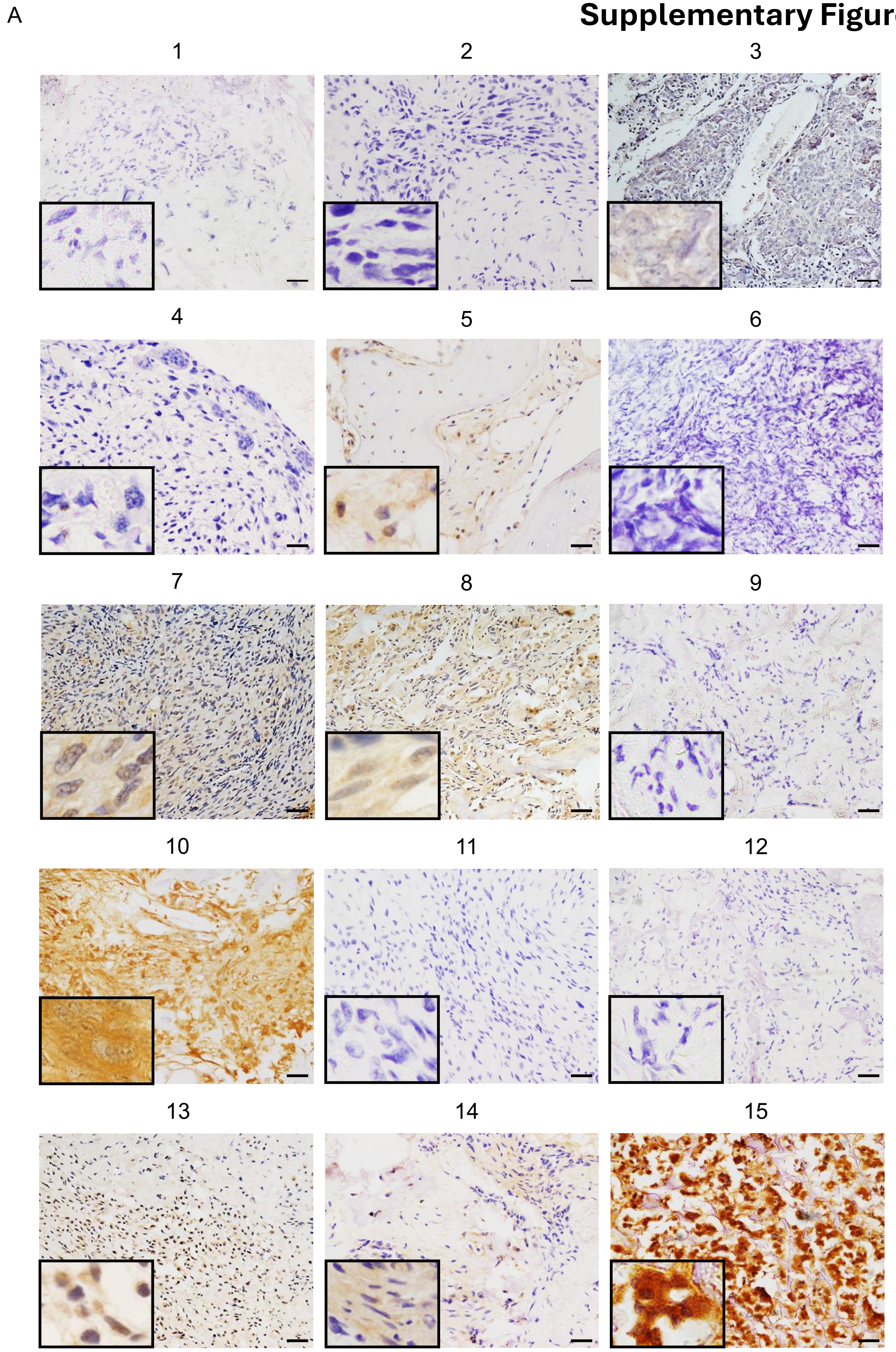

Supplementary Figure 6.

A

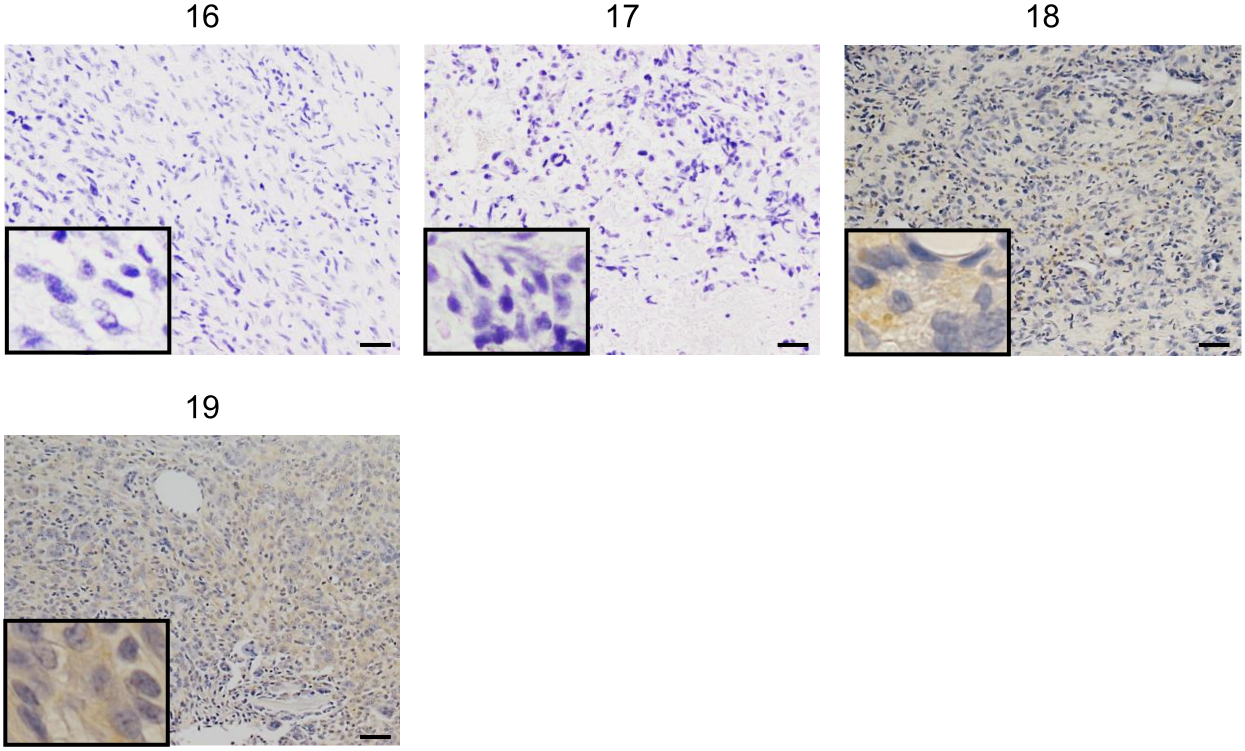

B

Case No.	Intensity
1	0
2	0
3	1
4	0
5	1
6	0
7	1
8	1
9	0
10	2
11	0
12	0
13	1
14	2
15	2
16	0
17	0
18	1
19	1

C

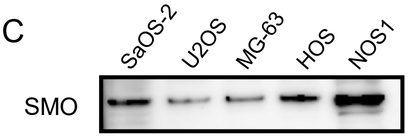

Supplementary Fig. 6 Expression of Hedgehog signaling genes in the clinical samples and cells of human osteosarcoma
(A) Immunohistochemistry analysis of 19 human clinical samples, with GLI1 expression detected in 10/19 samples. Scale bar: 50 µm
(B) Histological scoring was performed by two blinded pathologists: 0 = negative, 1 = weakly positive, and 2 = strongly positive
(C) Protein expression of SMO in human osteosarcoma cell lines. Prior to using the SMO inhibitor vismodegib, we confirmed that SMO was expressed in each osteosarcoma cell line
