# Supplementary material for: Ldha Regulates Osteosarcoma Lung Metastasis through Hedgehog Signaling
Source: Cancer Res Commun. 2026 Jun 25;6(6):1495–508. doi: 10.1158/2767-9764.CRC-25-0163 (PMC13295448; doi:10.1158/2767-9764.CRC-25-0163)
Supplement: Supplementary Fig.12 — Full membrane images supporting Figs. 6A, 6G, and Supplementary Fig.6C [file crc-25-0163_supplementary_fig.12_suppsf12.pdf]

Supplementary Figure 12.

Fig. 1B

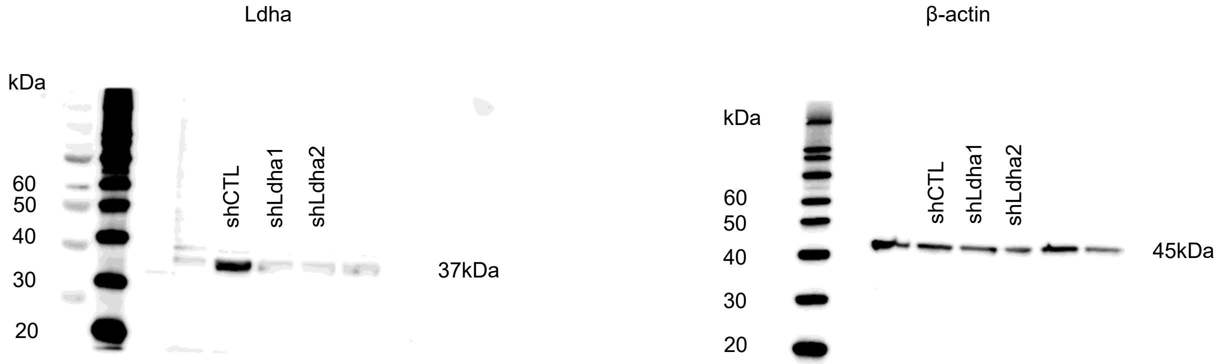

Fig. 3F

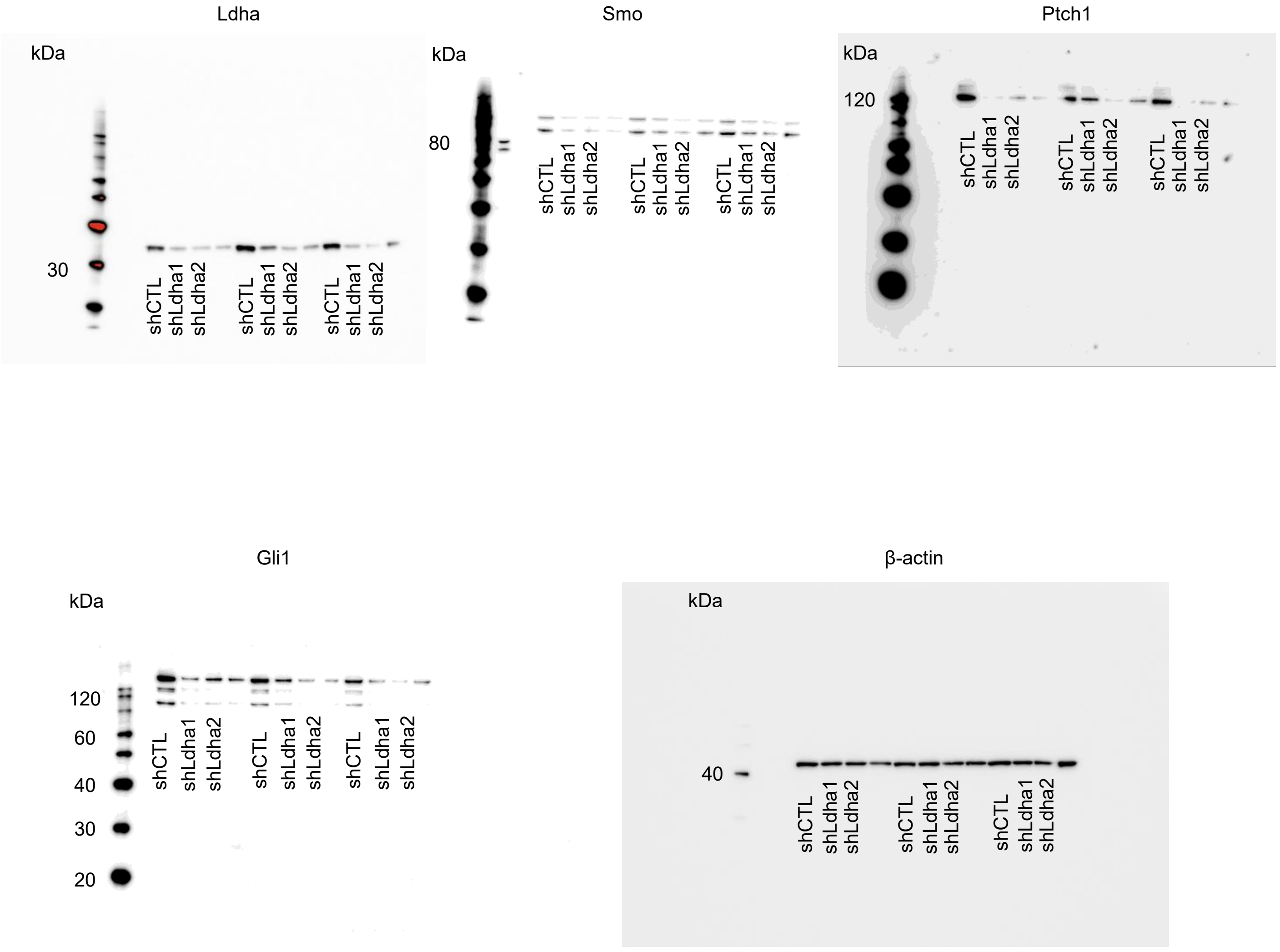

Supplementary Figure 12.

Fig. 6A

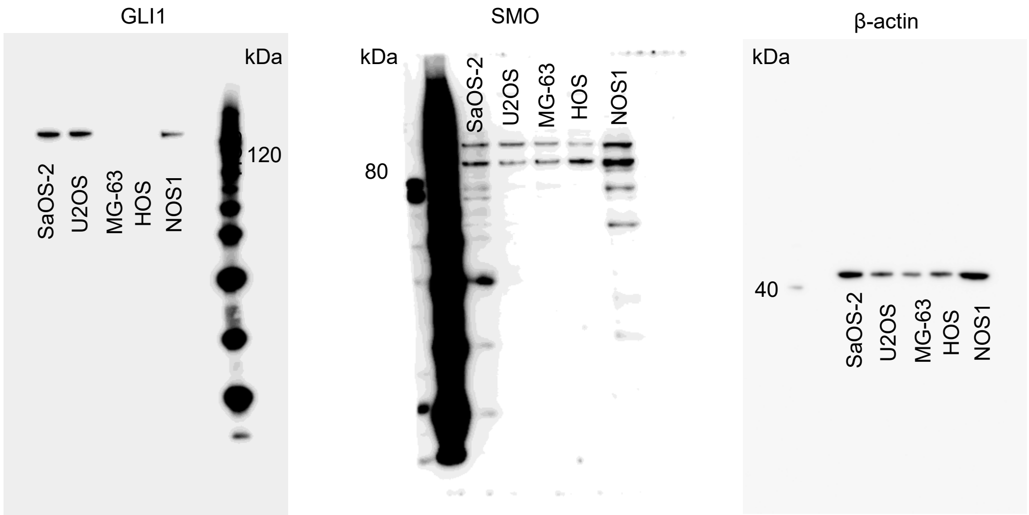

Fig. 6G

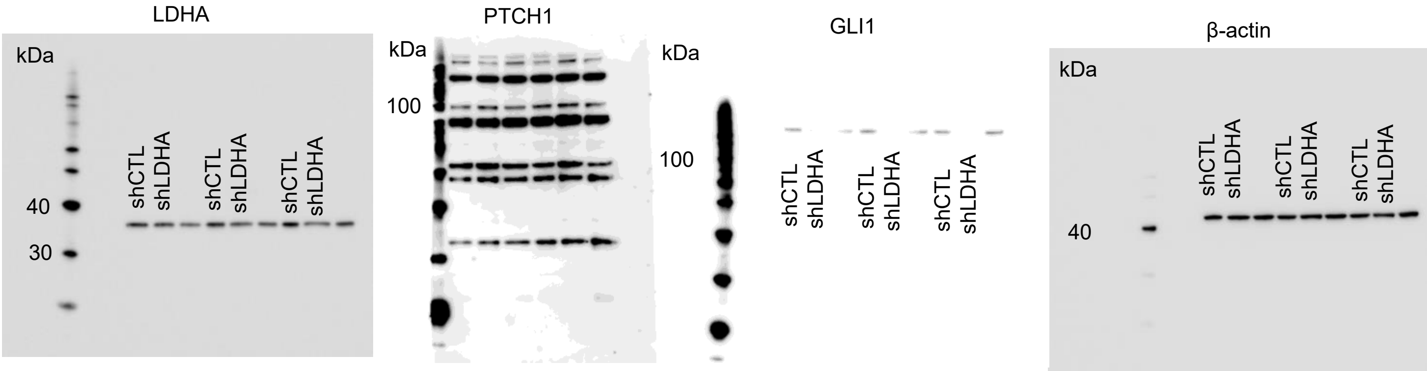

Supplementary Fig. 6C

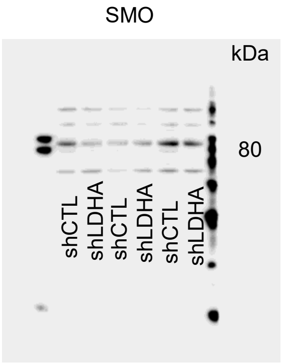

Supplementary Fig. 12 Full membrane images supporting Figs. 1B, 3F, 6A, 6G, and Supplementary Fig.6C
